# Clinical and Molecular Genetic Characterization of Landau Kleffner Syndrome: An Observational Cohort and Experimental Study

**DOI:** 10.1002/ana.27306

**Published:** 2025-09-13

**Authors:** Adeline Ngoh, Maria Clark, Rebecca Greenaway, Xiumin Chen, Kimberley M. Reid, Katy Barwick, Esther Meyer, Dale Moulding, Natalie Trump, J. Helen Cross, Sean D. Fraser, Lachlan de Hayr, Dimitri M. Kullmann, Joseph W. Lynch, Robert J. Harvey, Manju A. Kurian

**Affiliations:** ^1^ Developmental Neurosciences, UCL Great Ormond Street Institute of Child Health Zayed Centre for Research into Rare Disease in Children London UK; ^2^ Paediatric Neurology, KK Women's and Children's Hospital Singapore Singapore; ^3^ Department of Neurology Great Ormond Street Hospital London UK; ^4^ Department of Psychology Great Ormond Street Hospital London UK; ^5^ Queensland Brain Institute, The University of Queensland Brisbane Queensland Australia; ^6^ Developmental Biology and Cancer Programme UCL Great Ormond Street Institute of Child Health London UK; ^7^ School of Health University of the Sunshine Coast Sippy Downs Queensland Australia; ^8^ National PTSD Research Centre, UniSC Thompson Institute Birtinya Queensland Australia; ^9^ UCL Queen Square Institute of Neurology London UK

## Abstract

**Objective:**

Landau–Kleffner syndrome (LKS), is a rare, poorly‐understood epileptic encephalopathy with spike–wave activation in sleep associated with mutations in *GRIN2A*, encoding the N‐Methyl‐D‐Aspartate receptor (NMDAR) GluN2A subunit. Physicians rely on empirical treatments, with scarce information on treatment efficacy and outcomes. This study aims to improve the understanding and clinical management of LKS.

**Methods:**

Fifty‐two patients with LKS were recruited via one quaternary referral center. Case‐notes review delineated clinical features, long‐term outcomes, and prognostic factors. Generalized estimating equations were used to determine the longitudinal association among electroencephalogram abnormalities, steroid therapy, and neuropsychological findings. After genetic screening, the impact of identified *GRIN2A* missense variants on NMDAR function was assessed using homology modeling, cell‐surface trafficking assays, and electrophysiology in artificial synapses. Whole exome/genome sequencing was performed on *GRIN2A*‐negative patients to identify novel gene associations.

**Results:**

LKS is complex with significant clinical and genetic heterogeneity. Besides speech and language impairment, many patients had other co‐morbidities and almost half have long‐term disability. Early age at disease onset was associated with worse outcomes. There was no reliable correlation between electroencephalogram findings and developmental scores. Steroid therapy improved language outcomes independently of electroencephalogram findings. *GRIN2A* mutations were identified in 15.5% of the cohort. Likely pathogenic variants in *GABBR2*, *SCN1A*, *TRPC1*, *ERRFI1*, *CTXN3*, *IRX6*, and *IQCA1* were identified in 7 *GRIN2A‐*negative individuals.

**Interpretation:**

For LKS, early intervention is important for long‐term outcomes. Furthermore, management should not be based solely on electroencephalogram findings. Genetic and functional investigations offer insights into disease pathophysiology and facilitate development of future targeted therapies. ANN NEUROL 2025;98:951–966

It is well recognized that the cost of epilepsy to an individual lies not just in seizure burden but also in the associated co‐morbidities. The term “epileptic encephalopathy (EE)” was coined in 2001 by the International League Against Epilepsy (ILAE) to delineate conditions in which the epileptiform abnormalities themselves contribute to “progressive disturbance in cerebral function” that in turn results in these co‐morbidities.^1^ This term has since been revised a number of times. In the 2017 classification, in recognition that many childhood epilepsy syndromes traditionally viewed as EEs are associated with genetically determined developmental conditions, the terms “developmental and/or epileptic encephalopathy” were proposed.^2^ For children with developmental and epileptic encephalopathy (DEE), it can be difficult to distinguish how much of the encephalopathy or neuropsychological component is attributed to underlying developmental processes and how much is due to epileptic activity. This distinction has important treatment implications as over‐ascribing neuropsychological difficulties to seizure activity – when it may be due to genetically encoded perturbations in brain development – can result in unjustified over‐treatment.

Landau Kleffner syndrome (LKS) is a rare EE characterized by speech and language regression in association with a seizure disorder. In the latest ILAE classification of epilepsy syndromes, the eponym LKS continues to be recognized as a distinct subtype of epileptic‐encephalopathy with spike‐wave activation in sleep (EE‐SWAS) where regression affects primarily language skills, along with acquired auditory agnosia.^3^ In a Japanese epidemiology study, the incidence of LKS was estimated to be 1 in 978,000.^4^ Children present between the ages of 3 and 5 years old with acquired aphasia, and classical electroencephalogram (EEG) findings.^5^ Often, spike–wave discharges occupy more than 85% of the slow‐wave sleep record, that is, constitute a spike–wave index (SWI) of >85%, meeting classical criteria for electrical status epilepticus in sleep (ESES), otherwise known as “continuous spike‐waves in sleep” (CSWS).^6^

Eight to 20% of individuals with EE‐SWAS, including those with LKS, have been reported to have mutations in *GRIN2A*,^7^, ^8^, ^9^ encoding the GluN2A subunit of the N‐methyl‐D‐aspartate receptor (NMDAR).

LKS is a poorly understood EE associated with significant morbidity. In order to better delineate LKS, we undertook this study reporting the largest cohort of patients with LKS managed at a single quaternary pediatric neurology referral center. In this study, we have: (1) identified key clinical features and co‐morbidities; (2) assessed language and long‐term outcomes; (3) identified key prognostic factors; (4) evaluated how EEG findings and steroid therapy impact neuropsychological impairment; (5) determined *GRIN2A* mutation frequency; (6) explored the effect of *GRIN2A* mutations on NMDAR cell‐surface trafficking and function, (7) analyzed genotype–phenotype correlations, and (8) identified novel gene associations in *GRIN2A*‐negative patients. Our study aims to improve both the fundamental scientific understanding and clinical management of this challenging disorder.

## Methods

### Patient Recruitment

Patients referred with LKS from January 1989 to June 2018 were recruited from a quaternary pediatric neurology service (Great Ormond Street Hospital, London, UK). Written informed consent was obtained from all participants/legal guardians, and the study was approved by the Local Institutional Review Board (13/LO/0168). Study inclusion criteria were as follows: (i) a speech and language led regression; and (ii) evidence of a seizure disorder around the time of regression (i.e., clinical seizures and/or consistent EEG findings). Patients who had long‐standing speech and language impairment *without* a clear history of regression and those who had clinical features more in keeping with a global pattern of regression were excluded.

### Patient Assessment

Clinical data were extracted from the patient case note review. A variety of developmental assessments were used (Supplementary Methods in Data S1). Language outcomes were classified as: average, or mild, moderate, or severe impairment, or no functional language, as defined in Supplementary Table S1. Non‐verbal skills were classified as: average or above average, and below average. A patient was classified as having below average non‐verbal skills if any assessment performed during the course of illness scored more than 1 standard deviation below the expected score for his/her age.

A sleep EEG, along with a neurocognitive/speech and language assessment, was usually performed at presentation. All neuropsychological assessments and sleep EEGs were repeated 6 weeks after treatment initiation and then as needed afterward. The association of EEG findings and steroid therapy with language and behavioral outcomes was assessed in a subset of patients who had at least one sleep EEG recording within 6 months of a formal assessment of language and cognition.

For this part of the study, all scores for receptive language, expressive language, and non‐verbal skills were converted to developmental quotients (DQs) for statistical analysis (see Supplementary Methods in Data S1). EEG results for this study were classified as: (1) EEG‐0 = no sleep activation; (2) EEG‐1 = sleep activation but does not meet criteria for ESES (< 85% SWI); and (3) EEG‐2 = ESES (>85% SWI). Behavioral difficulties were assessed with a Strengths and Difficulties questionnaire filled in by the parents. Behavioral difficulties were categorized as: (1) no problem or manageable difficulties, and (2) severe problems defined as significant difficulties requiring medication or inpatient admission.

A proportion of the cohort who were diagnosed as children, but had reached adulthood at the time of this study (>18 years of age), were contacted to ascertain their level of independence, level of speech and language difficulties, and seizure burden. Outcomes as adults were classified as: (1) independent with mild or no language difficulties; (2) independent with significant language difficulties; and (3) dependent (Supplementary Table S2). The placement of patients into these categories was agreed upon unanimously by 3 independent clinicians.

### Statistical Analysis

The Statistical Product and Service Solutions (SPSS) statistical program was used to derive statistical values including frequency, mean values, standard deviation, and range. Fisher's exact test was used to compare discrete variables and 1‐way analysis of variance (ANOVA) with Bonferroni correction was used to compare continuous variables. Generalized estimating equations were used to explore the relationships between longitudinal measurements of EEG, steroid use, and patient psychometrics. For all analyses, a *p* value of < 0.05 was designated as the cutoff for statistical significance.

### Molecular Genetic Analysis

Lymphocytic deoxyribonucleic acid (DNA) was extracted from peripheral blood. The frequency of *GRIN2A* variants within the cohort was determined via Sanger sequencing, next‐generation DNA sequencing, and multiplex ligation‐probe amplification (MLPA) techniques. Some patients had *GRIN2A* screened via clinical epilepsy gene panel testing (see Supplementary Methods in Data S1). *GRIN2A‐*negative patients had DNA samples sent for triome‐whole exome sequencing (WES) or whole‐genome sequencing (WGS) at Beijing Genomics Institute, Beijing, Hong Kong (see Supplementary Methods in Data S1). Mapping of reads, calling, and recalibration of variant calls were performed using the GOSgene bioinformatics pipeline.^10^ The Qiagen Ingenuity Variant analysis platform was used to filter out low confidence variants, common variants, and likely benign variants (see Supplementary Methods in Data S1); and then carry out a comprehensive search strategy for novel gene associations. This search strategy included: (1) identifying variants within a panel of known epilepsy genes and previous genes reported in association with EE‐SWAS (EASD; Supplementary Table S3); and (2) performing individual familial trio analysis with *de novo*, recessive, and X‐linked recessive models of inheritance. Alamut Visual software (https://www.interactive-biosoftware.com) was used to annotate and prioritize variants. All families who underwent WGS were screened for structural variants using LUMPY.^11^ Other families had copy number variant (CNV) screening via single nucleotide polymorphism array analysis. Significant variants in candidate genes were verified with Sanger DNA sequencing. The American College of Medical Genetics and Genomics (ACMG) guidelines were used to help interpret sequence variants.^12^

### Molecular Modeling

Molecular modeling was performed using the crystal structure of the human GluN1‐GluN2A NMDAR or γ‐aminobutyric acid B receptor (GABA_B_R) obtained from the protein structure databank (PDB), 7EU7 (GluN1‐GluN2A NMDA receptor in complex with S‐ketamine, glycine, and glutamate) and 4PAS (heterodimeric coiled‐coil structure of human GABA_B_R). NMDAR and GABA_B_R structures were visualized using the UCSF ChimeraX molecular visualization program.^13^ Amino acid substitutions were modeled using the swapaa command, taking into account the highest rotamer prevalence (Dunbrack backbone‐dependent rotamer library),^14^ the highest number of H‐bonds, and the lowest clash score.

### In Vitro *Overexpression Cellular Model*

An overexpression human embryonic kidney 293 (HEK293) cellular model was used to study the effect of identified GluN2A missense variants on recombinant messenger ribonucleic acid (mRNA)/protein levels, and cell‐surface trafficking of GluN1/GluN2 NMDARs (see Supplementary Methods in Data S1).

### Electrophysiology

HEK293 cells were transfected with plasmids containing human GluN1 and wild‐type or mutant human GluN2A subunits, enhanced green fluorescent protein (pEGFP) and mouse neuroligin 1B at a ratio of 1:1:0.5:1, using a calcium‐phosphate co‐precipitation protocol, as previously described.^15^ Rat cortical neurons were cultured for three to 4 weeks before freshly transfected HEK293 cells were plated onto the neurons. Artificial synaptic connections formed within 24 hours and excitatory post‐synaptic currents (EPSCs) in HEK293 cells were recorded by a whole‐cell patch clamp 2 to 5 days later (see Supplementary Methods in Data S1).

### Role of Funding Sources

Funding supported patient recruitment, data collection, analysis, and interpretation. However, the funders had no role in study design, data collection and analysis, decision to publish, or preparation of the manuscript.

## Results

### Clinical Characterization of the Patient Cohort

**FIGURE 1:**
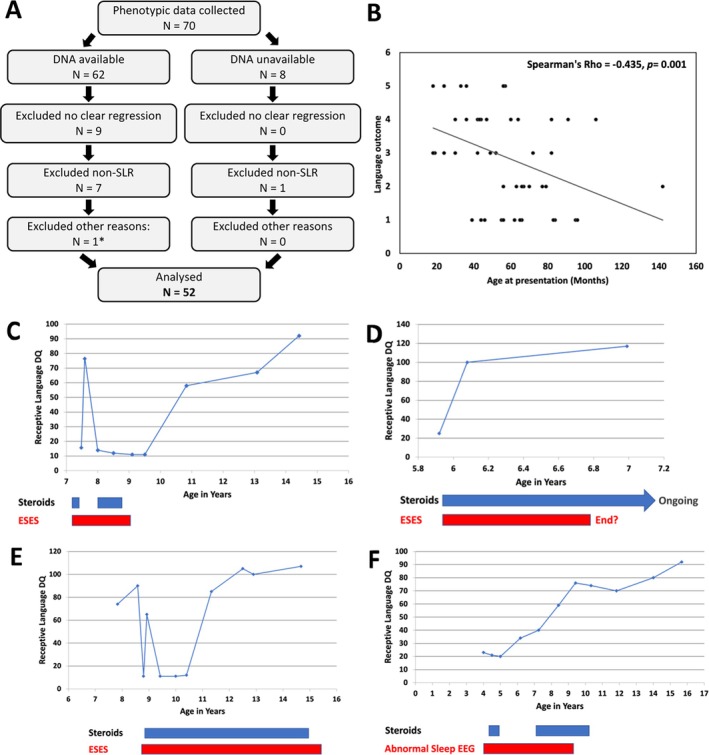
Characterization of the LKS patient cohort. (A) Patients included in cohort study. *One patient was later found to be NMDAR antibody positive, and was excluded from the study. (B) Correlation between age at regression and language outcomes. Language outcomes grouped into: 1 = normal/average language skills; 2 = mild impairment; 3 = moderate impairment; 4 = severe impairment; and 5 = no functional language. (C–F) Profile of individual patients – correlating receptive language DQ with timing of ESES and steroid therapy. (C) Improvement on steroids despite ESES, and then relapse. (D) Remarkable improvement maintenance despite ESES. (E) Improvement and deterioration not correlating with steroids or ESES. (F) Improvement despite abnormal sleep EEG with activation in slow wave sleep. DQ = developmental quotient; EEG = electroencephalogram; ESES = electrical status epilepticus in sleep; LKS = Landau–Kleffner syndrome; N = number of patients; NMDAR = N‐Methyl‐D‐Aspartate receptor; SLR = speech and language regression. [Color figure can be viewed at www.annalsofneurology.org]

Phenotypic data were collected for 70 patients. Eighteen patients were excluded based on study inclusion/exclusion criteria (Fig 1A). Data from 52 patients were analyzed.

#### Clinical Features

In this study, 59.6% of this cohort were male patients. Combining the results of our study with available results from previous studies in Table 1, overall, 61.6% ± 7.8% of reported patients were male patients. The mean age at language regression for our cohort was 4 years 9 months ± 2 years (range = 1 year and 6 months to 11 years and 10 months). The age of onset of aphasia has been relatively consistent across all cohort studies over the last 4 decades, with a mean age of presentation from 3.5 to 5 years of age. Nearly half the patients in our cohort (42.3%) have a positive family history of seizures or speech disorders (see Table 1).

**TABLE 1:** Data Collected in Comparison with Previously Reported Cohort Studies

Year	1980^18^	1989^19^	1992^20^	1993^21^	1994^22^	1999^5^	2001^23^	2009^17^	2011^24^	2014^16^	This Study
No.	9	7	6	6	12	11	17	7	19	29	**52**
Male	33.3%	85.7%	33.3%	NR	75%	63.6%	35%	100%	NR	68.9%	59.6% ± 13.3%^d^
Positive FH	22.2%	NR	0%	NR	NR	45.5%	11.7%	NR	NR	NR	42.3% ± 13.4%^d^
SD	0%	14.2%	0%	0%	75%	27.3%	23.5%	NR	0%	65.5%	28.8% ± 12.3%^d^
Mean SLR age (range)	5 yr 9 mo (3 yr–9 yr)	5 yr (3 yr–8 yr)	4 yr 8 mo (3 yr 1 mo–6 yr 8 mo)	4 yr 9 mo (2 yr 0 mo–6 yr 10 mo)	4 yr 10 mo (4 yr–5 yr 6 mo)	3 yr 5 mo (1 yr 6 mo–5 yr 8 mo)	4 yr 8 mo (2 yr 1 mo–7 yr)	4 yr 6 mo (3 yr–9 yr)	3 yr 7 mo (1 yr 5 mo–6 yr)	5 yr (2 yr–9 yr)	4 yr 9 mo ± 2 yr^e^ (1 yr 6 mo–11 yr 10 mo)
Sz	66.7%	85.7%	100%	66.7%	58%	81.8%	88.2%	85.7%	47%	79.4%	84.6% ± 9.8%^d^
ESES (SA)	NR	NR	75.0%^b^	NR	NR	100%	NR (100%)	28.6% (71.4%)	NR (89.5%)	51.7% (41.3%)	55.8% ± 13.4%^d^ (25.0% ± 11.8%^d^)^b^
< ave NVS	0%	28.6%	0%	NR	50%	81.8%	11.8%	NR	15.7%	48.3%	46.2% ± 13.6%^d^
BD	44.4%	71.4%	0%	83.3%	75%	72.7%	52.9%	42.8%	89.5%	65.5%	76.9% ± 11.5%^d^
Lang. OC at last FU	NL: 44.4% MI: 11.1% MDI: 44.4%	NL: 28.6% MI/MDI: 14.3% SI/NS: 42.9%	NL: 16.7% MI: 33.3% MDI: 33.3% SI: 16.7%	MI: 50.0% MDI: 16.7% SI: 33.3%	NL: 25% MI/MDI: 57.9% SI: 16.6%	NL: 18.2% MDI: 45.5% SI: 36.3%	NL: 17.6% MDI: 23.5% SI/NS: 23.5%^a^	NL: 14.3% MI‐SI: 85.8% (42.9% PR)	NL: 21% MDI: 58% SI/NS: 21%	NL: 27.5% MI‐SI: 73.5%	NL: 26.9% ± 12.1%^d^ MI: 15.4% ± 9.8%^d^ MDI: 19.2% ± 10.7%^d^ SI: 23.1% ± 11.5%^d^ NS: 15.4% ± 9.8%^d^
Sz at last FU	0%	14.3%	16.7%	NR	NR	0%	NR	28.6%	0%	10.3%	21.2% ± 11.1%^d^
OC at >18 yr	N: 7 NL/I: 57.1% MI/I 14.3% MDI/I: 28.6%	N: 7 I: 42.9% D: 57.1%	N: 1 I: 100%	N: 0	N: 3 NL: 66.6% MDI: 33.3% I: NR	N: 1 SI: 100% I: NR	N: 0	N: 4 NL/I: 25% MI – SI/D: 75% (25% PR)	N: 4 NL/I: 75% MDI/I: 25%	N: 13 NL/I: 46.2% MI‐MDI/I: 30.8% SI/D: 23.1%	N: 25^c^ NL‐MI/I: 56% ± 19.5%^d^ MDI‐SI/I: 28% ± 17.6%^d^ D: 16% ± 14.4%^d^

ADHD = attention deficit hyperactivity disorder; Agg = aggression; ASD = autistic traits; ave = average; a/w = associated with; BD = behavioral disorder; corr = correlated; D = dependent; EEG = electroencephalogram; ESES = electrical status epilepticus in sleep (85% of spike wave index); Positive FH = positive family history of seizures or speech and language impairment; FU = follow‐up; mo = months; HA = hyperactivity; I = independent; Lang. = language; MI = mild speech impairment; MDI = moderate speech impairment; N = number of patients; NL = normal language; NR = not reported; NS = no speech; NVS = non‐verbal skills; OC = outcome; PR = partial remission; SA = sleep activation (< 85% spike wave index); SD = pre‐existing speech delay; SI = severe speech impairment; SLR = speech and language regression; Sz = clinical seizures; yr = years.

^a^ Remainder: not reported.

^b^ Two of 6 (33.3%) for Paqueir et al and 9 of 52 (17.3%) for this present study did not have sleep EEG data available, calculated percentages are based on patients with sleep EEG data available.

^c^ Formal speech and language assessments were not repeated.

^d^ The 95% confidence interval.

^e^ Standard deviation.

In this study, 84.6% of our patients had epileptic seizures. Including results from previous studies, overall, 77.1% ± 6.2% of patients had clinical seizures. The most frequently reported seizure type in our cohort was focal motor seizures occurring from sleep. This is consistent with previous reports.^5^, ^16^, ^17^ Other seizure types include absence, atypical absence, myoclonic, atonic, and generalized tonic–clonic seizures. 55.8% of our cohort had ESES (SWI >85%), a further 25% had SWI of 30% to 81%. Nine of 52 patients (17.3%) did not have available sleep EEG recordings. The clinical features of these patients are detailed in Supplementary Table S4. They were diagnosed with LKS based on a clear speech and language regression in association with epilepsy, in accordance with Landau and Kleffner's original description of the syndrome. We note that, currently, LKS is classified as a sub‐type of DEE‐SWAS.^3^ As such, these patients may not strictly meet a diagnosis of LKS today.

Of our cohort, 28.8% had pre‐existing speech delay (other cohorts = 0%–75%). Overall, including available data from previous studies, 30.3% ± 7·0% of the patients had pre‐existing speech delay. There were 44.2% of this cohort that had below average non‐verbal skills (other cohorts = 0%–81.8%). Overall, 37.0% ± 7.4% of reported patients have below average non‐verbal skills. Behavioral difficulties are also common in LKS, occurring in 76.9% of this cohort. Overall, 69.9% ± 7.0% (114/163) of reported patients have behavioral difficulties. In our cohort, aggression is most commonly reported (54%), followed by hyperactivity (38%). Although motor difficulties have rarely been reported in previous studies of LKS, in our cohort, 28.8% had motor difficulties. These comprised fine motor deficits (11.5%), dyspraxia (11.5%), gait impairment (3.8%), and dyskinesia (1.9%).

#### Language Outcomes

Classification of language outcomes varied among different studies. There were 26.9% (95% confidence interval = 14.8% to 39.0%) of our cohort that had recovered language at their last assessment, scoring within the average range expected for their age compared to 14.3% to 44.4% in previous studies. Overall, including previous studies, 24.0% ± 6.3% of reported individuals recover normal language. The remaining had some degree of language impairment ranging from mild language impairment to no functional language at last follow up.

#### Factors Affecting Language Outcomes

To determine what factors might influence language outcome in our cohort, we looked for significant differences in all collected data variables across the different language outcome groups (Supplementary Table S5). The only data variable that differed significantly across the different language outcome groups was age at onset of regression (AOR). Further analysis with Spearman's correlation revealed a highly significant moderate negative correlation between AOR and language outcome (Spearman rho = −0.435, n = 52, *p* = 0.001; Fig 1B). The proportion of patients with clinical seizures and the proportion of patients with ongoing ESES was not significantly different across the different language outcome groups.

#### Effect of EEG and Steroid Therapy on Patient Outcomes

While collecting clinical data, we often observed that the trajectory of recovery for individual patients did not correlate well with steroid therapy or EEG findings (Fig 1C–F). We therefore performed a sub‐study on 49 patients in the cohort to examine more closely the longitudinal association of EEG findings and steroid therapy with language outcome, non‐verbal skills, and behavior. All patients in this part of the study had an EEG performed within 6 months of the date of neuropsychological assessment (57% within the same month, and 81% within 3 months). EEG and neuropsychological assessments were closely coupled with clinical status. These were conducted more frequently during acute illness or relapse, and less frequently during periods of clinical stability. Data on collected variables and regression coefficients are presented in Table 2. Other data are presented in Supplementary Table S6.

**TABLE 2:** Effects of Steroids and ESES on Language, Cognition, and Behavior

	Regression Coefficients (βs)			Odds Ratios
	Expressive Language	Receptive Language	Non‐Verbal Intelligence	Extreme Behavior
Age at assessment	0.062**	0.052**	−0.015*	0.845
Steroids	0.321**	0.282**	0.001	0.678
Sleep spike–wave >85%	−0.288**	−0.282*	−0.032	2.812*
Sleep spike–wave < 85%	−0.014	−0.106	0.096	3.077*

Cell entries are regression coefficients (βs) based on generalized estimating equations models. Within 6 months of a sleep EEG, 48 patients had a total of 137 expressive DQ assessments, 49 patients had a total of 142 receptive DQ assessments, 45 patients had a total of 105 non‐verbal DQ assessments, and 49 patients had a total of 159 behavioral assessments.

DQ = developmental quotient; EEG = electroencephalogram; ESES = electrical status epilepticus in sleep.

* *p* < 0.05.

** *p* < 0.01.

Important predictors for expressive DQ (EDQ) and receptive DQ (RDQ) are age at assessment and steroids. Older patients and those on steroids achieved higher scores at assessment. EEG improves the fit of the model. For EDQ, EEG is a significant predictor at *p* = 0.048. This is due to a significant difference between EEG‐0 and EEG‐2. EEG‐1 does not have a significant effect. For RDQ, the effect of EEG is not significant (*p =* 0.096). For both EDQ and RDQ, the interaction between EEG and steroids is not significant and adding the interaction between EEG and steroids does not improve the fit of the model. The only important predictor for non‐verbal DQ is age at assessment. Steroids and EEG do not appear to have a significant effect. EEG (any sleep activation) is the only significant predictor for severe behavioral problems; steroid therapy is not a significant predictor.

#### Adult Long Term Outcomes

Twenty‐five of 52 individuals in our cohort are now over the age of 18 years. Fifty‐six percent of these individuals are independent with no or mild language difficulties. Twenty‐eight percent are independent but continue to have significant language difficulties. A further 16% continue to be dependent.

### *GRIN2A* Analysis With Comparison of Positive and Negative Individuals

Genomic DNA was available for 45 of the 52 patients with LKS. Seven patients (15.5%) were found to have *GRIN2A* mutations (Table 3) which were identified through diagnostic panel testing (n = 2), Sanger sequencing (n = 4), diagnostic microarray, and MLPA (n = 1). Of these, 4 are previously unpublished (p.R518C, p.V430Qfs*18, p.A593Kfs*62, and p.H1071Lfs*33). Two were inherited from unaffected parents; both of these parents have not had EEGs or formal assessments for speech and language. Notably, incomplete penetrance has previously been reported for inherited *GRIN2A* variants within EE‐SWAS.^7^, ^8^, ^9^

**TABLE 3:** Mutations Identified in *GRIN2A* Encoding the NMDAR GluN2A Subunit

Mutation	R/N	Means of ID	Inh (Seg)	dbSNP	1000G MAF	GD AF	EVS	CADD	ProV	SIFT	PP
c.2041C>T p.Arg681*	R^9^	RSS	NPS	rs397518472	Abs	Abs	Abs	**44.0**	N.A.	N.A.	N.A.
c.1552C>T p.Arg518Cys	N	RSS	UF (No)	rs747838255	Abs	0.00041%	Abs	**27.1**	**−7.71**	**0.00**	**1.00**
c.1289_1290delGT p.Val430Glufs*18	N	RSS	UM (No)	Abs	Abs	Abs	Abs	N.A.	N.A.	N.A.	N.A.
c.1776_1777dupAA p.Ala593Lysfs*62	N	RSS	*De novo*	Abs	Abs	Abs	Abs	N.A.	N.A.	N.A.	N.A.
arr 16p13.2p13.13 (9,287,840–10,889,600) x1	R^28^	MLPA and SNP array	*De novo*	rs397518469	Abs	Abs	Abs	N.A.	N.A.	N.A.	N.A.
c.1553G>A p.Arg518His	R^8^	Diagnostic gene panel	*De novo*	rs397518470	Abs	Abs	Abs	**28.0**	**−4.82**	**0.00**	**1.00**.
c.3212_3221del p.His1071Leufs*33	N	Diagnostic gene panel	AM (Yes)	Abs	Abs	Abs	Abs	N.A.	N.A.	N.A.	N.A.

CADD predicts a continuous phred‐like score that ranges from 1 to 99, with *higher values* indicating more deleterious cases. If the PROVEAN score is equal to or below a predefined threshold (−2.5), the protein variant is predicted to have a deleterious effect. The PolyPhen‐2 score ranges from 0.0 (tolerated) to 1.0 (deleterious). A SIFT score is a normalized probability of observing the new amino acid at that position, and ranges from 0 to 1. A value of between 0 and 0.05 is predicted to affect protein function. Note that both p.Arg518Cys and p.Arg518His are predicted to be deleterious or non‐tolerated by all 4 programs.

Abs = absent; AF = allele frequency; AM = affected mother; CADD = combined annotation dependent depletion score; dbSNP = single nucleotide polymorphism database; EVS = exome variant server; GD = genome aggregation database; ID = identification; Inh = inherited; MAF = minor allele frequency; N = novel; N.A. = not applicable; NPS = no parental samples; PP = Polyphen score; ProV = PROVEAN score; R = reported; Seg = segregates; UF = inherited from unaffected father; UM = inherited from unaffected mother; 1000G = 1000 Genomes project database.

We compared clinical variables between *GRIN2A‐*positive and *GRIN2A*‐negative LKS individuals in our cohort to look for phenotypic differences between these groups (Supplementary Table S7). However, the relatively small number of *GRIN2A* positive individuals in our cohort limits the strength of this analysis. To achieve better statistical comparison, we combined our data with that of all other patients with LKS with *GRIN2A* genotype reported in literature to date (Supplementary Tables S8–S10). Analysis of this larger cohort demonstrated that patients with *GRIN2A‐*positive LKS are statistically more likely to have a positive family history (*p* = 0.021), prior delay in speech development (*p* = 0.029), and motor difficulties (*p =* 0.019). They are less prone to significant behavioral difficulties (*p =* 0.033). There was no significant difference in language outcomes between the *GRIN2A* positive and negative groups both within our cohort and after incorporation of data from published literature.

### Effects of *GRIN2A* Mutations on NMDAR Structure and Function

**FIGURE 2:**
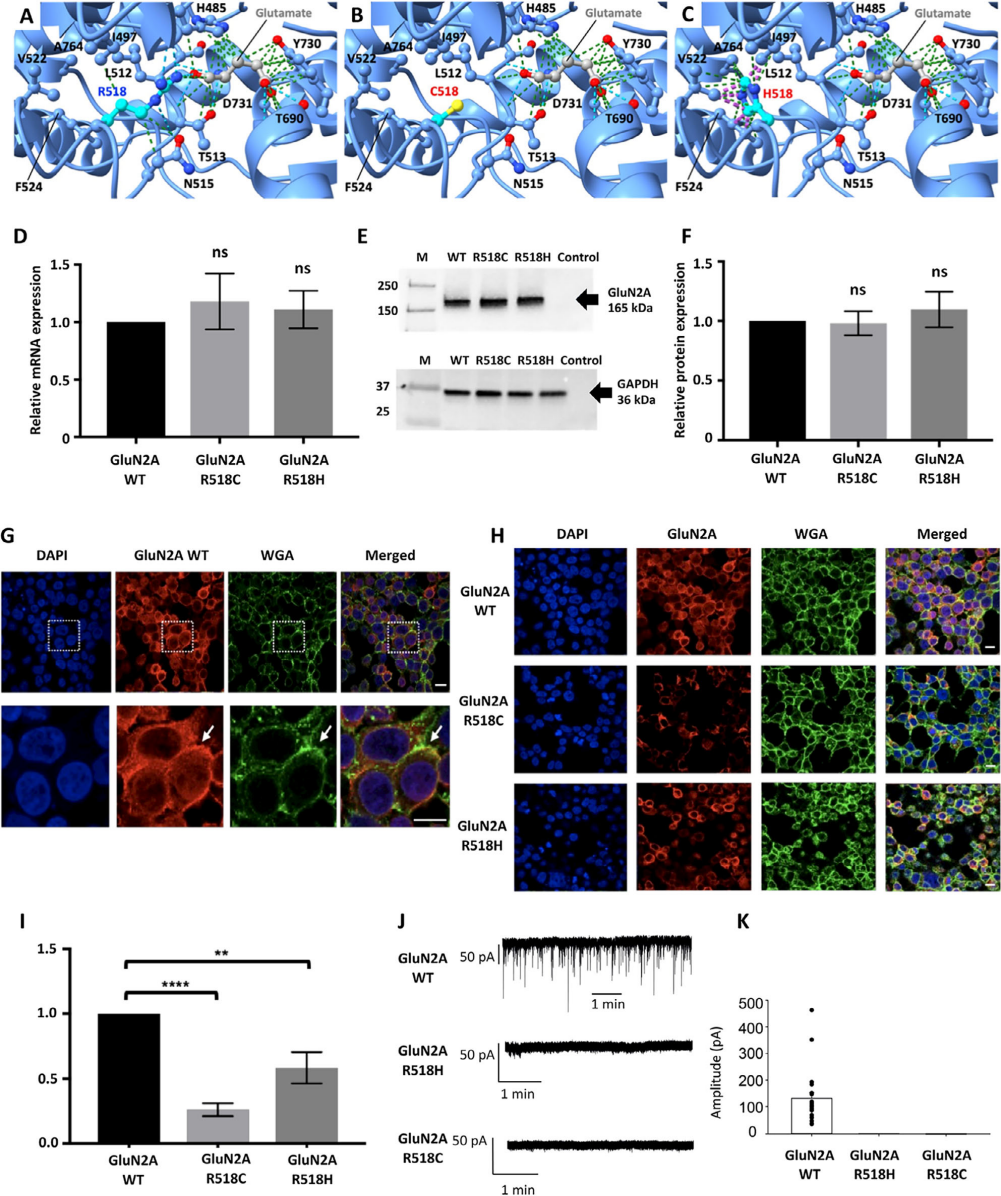
Molecular modelling and effects of GluN2A missense variants on recombinant mRNA protein levels and cell‐surface trafficking. (A–C) R518 is a key agonist binding residue. (A) NMDAR agonist‐binding domain from PDB 7EU7 showing L‐glutamate making contacts (green) with GluA2 H485, S511, L512, T513, T690, Y730, D731, and H‐bonds (blue) with T690, T513, and R518. The R518 makes contacts with I497 and N515, and H‐bonds with G486 and T513. (B) The shorter side chain of C518 loses contacts with L‐glutamate/I497/N515 and H‐bonds with G486/T513, but makes one new contact with F524. (C) H518 loses all contacts with L‐glutamate/N515 and H‐bonds with G486/T513, and has significant clashes (purple) with I497, V522, F524, and A764. (D) Relative gene expression (fold‐change) for GluN2A variants R518C and R518H normalized to wild‐type GluN2A (data derived from triplicate measurements). (E) Representative Western blot of HEK293 whole‐cell lysates probed with anti‐GluN2A antibody (top) and anti‐GAPDH antibody as a loading control (bottom), with (F) plot of protein expression for GluN2A^R518C^ and GluN2A^R518H^ variants relative to wild‐type GluN2A, normalized to GAPDH (average results from 3 independent transfections). (G) Representative confocal microscopy image of HEK293 cells co‐transfected with wild‐type GluN1 and wild‐type GluN2A (×63 magnification) enlarged to show co‐localization of surface GluN2A with membrane marker WGA (arrow). (H) Comparison of confocal microscopy images from HEK293 cells transfected with wild‐type GluN1 and either wild‐type GluN2A, or GluN2A^R518C^ and GluN2A^R518H^ variants (×63). (I) Quantification of relative fluorescence intensity of surface GluN2A^R518C^ and GluN2A^R518H^ compared to wild‐type GluN2A (average measurements from 3 independent transfections). (J) Properties of EPSCs mediated by di‐heteromeric GluN1/GluN2A, GluN1/GluN2A^R518H^ and GluN1/GluN2A^R518C^ NMDARs expressed in artificial synapses. (K) Mean amplitude of EPSCs from cells expressing the indicated subunit combinations. The n values for each observation were GluN1/GluN2A (n = 22), GluN1/GluN2A^R518H^ (n = 12), and GluN1/GluN2A^R518C^ (n = 18). Statistics undertaken using one‐way ANOVA in Prism 7.0 (GraphPad). ANOVA = analysis of variance; Con = non‐transfected HEK293 cells; EPSC = excitatory post‐synaptic currents; ns = not significant; NMDAR = N‐Methyl‐D‐Aspartate receptor; PDB = protein structure databank; WGA = wheat germ agglutinin; WT = wild‐type. **p = < 0.005, ****p = < 0.0001. [Color figure can be viewed at www.annalsofneurology.org]

Five of the *GRIN2A* mutations identified in our cohort (1 whole gene deletion, 3 intragenic frameshift deletions or insertions, and 1 nonsense mutation) are predicted to be inactivating protein truncating variants (PTVs). To elucidate the impact of the 2 identified missense variants (p.R518C and p.R518H), we investigated their effect on NMDAR protein structure, mRNA and protein levels, cell‐surface trafficking, and function using electrophysiology. R518 is a highly conserved residue across all NMDAR subunits, and is involved in agonist binding. Homology modeling shows that the replacement of the highly conserved arginine, which interacts with the acidic group of glutamate in the ligand‐binding site, by either cysteine or histidine, is likely to affect the agonist binding properties of GluN2A (Fig 2A–C). Real‐time polymerase chain reaction (RT‐PCR) performed in triplicate revealed no significant difference in mRNA levels when comparing wild‐type GluN2A with GluN2A^R518C^ and GluN2A^R518H^ in cells transfected with GluN1/GluN2A combinations (Fig 2D). There was also no significant difference in GluN2A total protein expression between wild‐type and mutant transfected cells (Fig 2E, F). However, immunofluorescence for cell‐surface localization revealed that both GluN2A^R518C^ and GluN2A^R518H^ have significantly reduced cell‐membrane expression compared to wild‐type GluN2A (Fig 2G–I).

Finally, we examined the effect of GluN2A^R518C^ and GluN2A^R518H^ variants on EPSCs mediated by GluN1/GluN2A NMDARs in artificial synapses. EPSCs mediated by wild‐type GluN1/GluN2A NMDARs were recorded in 40 of 50 cells over a total of 4 transfections (Fig 2J, K). We saw no evidence for EPSCs mediated by GluN1/GluN2A^R518C^ or GluN1/GluN2A^R518H^ NMDARs in a total of 25 cells each spread out over 4 transfections. Similarly, we saw no evidence for whole‐cell glutamate‐activated currents in HEK293 cells transfected with GluN1/GluN2A^R518C^ or GluN1/GluN2A^R518H^ combinations, indicating severe loss of agonist potency as well as reduced cell‐surface trafficking.

### Novel Gene Associations

**FIGURE 3:**
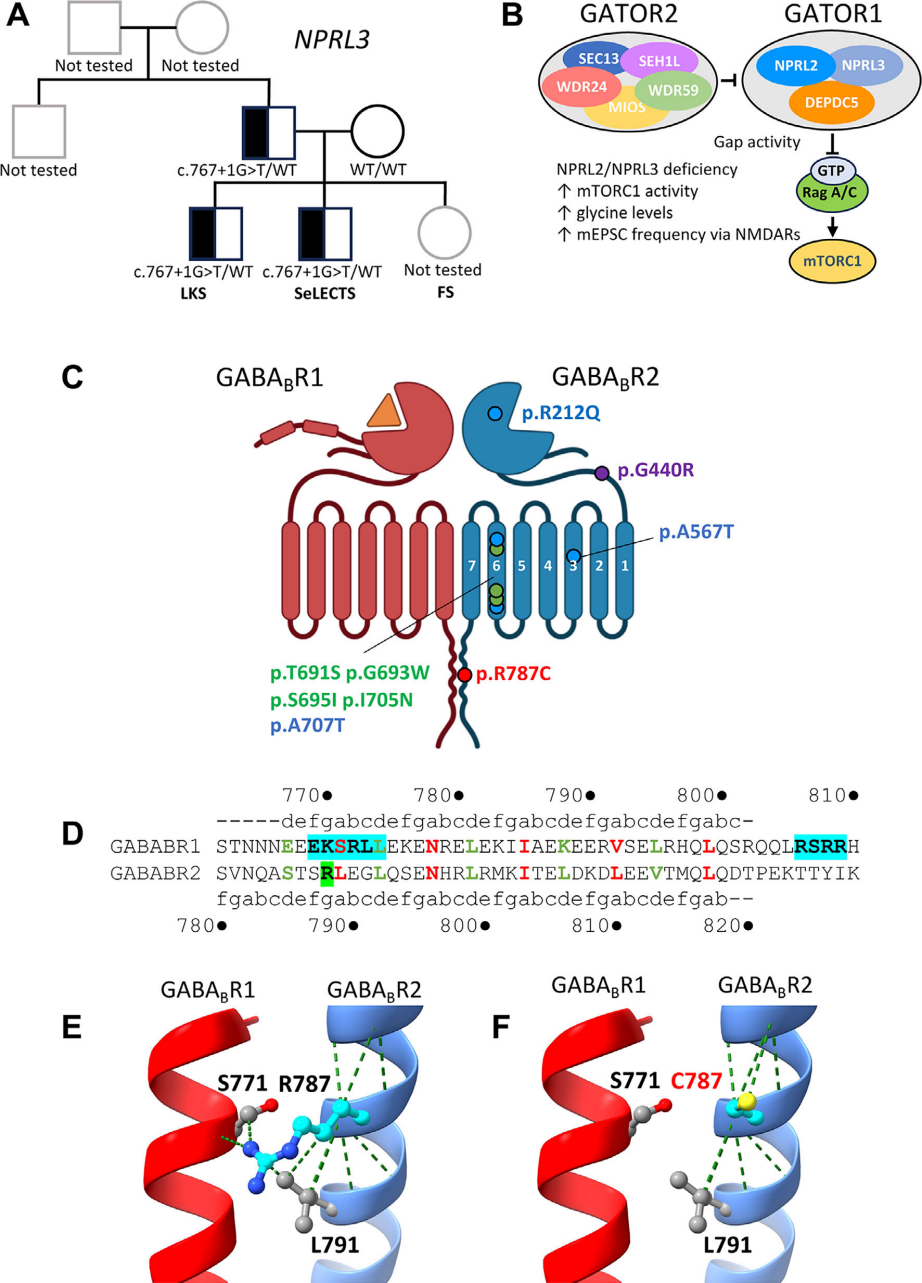
Novel gene associations. (A) Family pedigree for NPRL3 c.767 + 1G>T. (B) Proposed pathway linking NPRL3 deficiency to increased NMDAR activity. Defects in NPRL2/NPRL3, key components of the GATOR1 complex, result in increased mTORC1 activity, resulting in increased levels of the NMDAR co‐agonist glycine, which increases mEPSC frequency via NMDARs. (C) GABA_B_ receptor subunits and topology. GABA_B_R1 and GABA_B_R2 are composed of 3 domains: a long extracellular N‐terminal domain with a Venus flytrap domain, which contains the orthosteric binding site for GABA; a heptahelical transmembrane domain, and an intracellular C‐terminus. Missense mutations: red = LKS; green = developmental and epileptic encephalopathy; blue = neurodevelopmental disorder with poor speech and loss of hand skills (NDPLHS); purple = absence epilepsy and mild developmental delay. (D) The association between GABA_B_R1 and GABA_B_R2 is mediated by masking of: (i) a dileucine internalization signal (EKSRLL) and (ii) an ER retention signal (RSRR; cyan shading) in the cytoplasmic region of GABA_B_R1, facilitating cell‐surface expression of both subunits. A series of polar interactions buried within the hydrophobic core determines the specificity of heterodimer pairing. These hydrophobic residues include S771, L774, L781, I785, V792, and L795 of GABA_B_R1. Positions in GABA_B_R2 that are critical for the surface transport include L790, L791, L798, L805, L809, and V812. (E, F) GABA_B_R2 R787 forms contacts with critical residues L791 in GABA_B_R2 and S771 in GABA_B_R1 subunit. These contacts are lost upon substituting arginine 787 for cysteine (R787C), which is predicted to impair assembly and cell‐surface expression of the GABA_B_R1/GABA_B_R2 dimer. EPSC = excitatory post‐synaptic currents; ER = endoplasmic reticulum; FS = focal seizures; LKS = Landau Kleffner syndrome; NDPLHS = neurodevelopmental disorder with poor speech and loss of hand skills; NMDAR = N‐Methyl‐D‐Aspartate receptor; SeLECTS = self‐limiting epilepsy with centrotemporal spikes. [Color figure can be viewed at www.annalsofneurology.org]

For *GRIN2A*‐negative cases, 27 trios were sent for WGS. Due to budget constraints and as WGS was not as easily accessible early on in the study, 11 triomes were sent for WES. Identified likely pathogenic variants are presented in Table 4. The epilepsy gene panel search strategy identified *de novo* variants in two well‐known epilepsy genes: c.2359C>T (p.R787C), a novel variant in the GABA_B_R2 subunit gene (*GABBR2*), and c.748G>A (p.V250I), a previously reported variant in *SCN1A*.^25^, ^26^ A familial c.767 + 1G>T splice‐site variant in *NPRL3* (NPR3 Like, GATOR1 Complex Subunit) was also identified in a proband with LKS. His younger brother carried the same variant and had a diagnosis of self‐limited epilepsy with centrotemporal spikes (SeLECTS). This variant was inherited from their father who has no history of seizures or speech and language disorder and has not had an EEG. Their younger sister and paternal uncle also had seizures but their DNA samples were not available for testing (Fig 3A).

**TABLE 4:** Likely Pathogenic Variants Identified in *GRIN2A‐*Negative Individuals

Gene/Variant	Gene Function/Associated disorders	Inh	dbSNP/1000G MAF/GD/EVS	GVGD/Splicing prediction	CADD/SIFT	PP	ACMG
*GABBR2* NM_005458.8 c.2359C>T p.R787C Novel	Encodes: GABA_B_ receptor subunit 2. Function: mediates slow synaptic inhibition. **Disorders**: EIEE‐59, NPLHS, absence epilepsy with mild DtD^29^	DN	Abs/Abs/Abs/Abs	Class C0 (GV: 249.02 ‐ GD: 59.88)	32/Del 0.03	HD: PD 1.00 HV: PD 0.99	Likely Pathogenic PS2 + PM1 + PM2 + PP3
*SCN1A* NM_001165963.4 c.748G>A p.V250I ClinVar: 206,746	Encodes: Na_v_1.1 subunit of voltage‐gated sodium channels Function: generation of AP **Disorders**: GEFS+, DS, other epilepsy syndromes including: SeLECTs and DEE‐SWAS^−^ ^30^, ^31^, ^32^	DN	rs796052962/Abs/Abs/Abs	Class C25 (GV: 0.00 – GD: 28.68)	27/Del 0.00	HD: PD 0.992 HV: PD 0.992	Likely pathogenic PS2 + PM2 + PP2
*NPRL3* NM_001077350.3 c.767 + 1G>T ClinVar: 580,189	Encodes: nitrogen permease regulator 3 like protein, a part of the GATOR1 complex Function: regulation of neurogenesis, dendritic morphology, and LTP **Disorders**: FFEVF^27^	AS, UF	rs1567134495/Abs/Abs/Abs	MaxEnt: −100.0% NNSPLICE: −100.0% SSF: −100.0%	27.5/N.A.	N.A.	Likely pathogenic PVS1 + PM2 + PP5
*TRPC1* NM_001251845.2 c.961‐2A>C Novel	Encodes: tyrosine kinase receptor activated Ca^2+^ permeable non‐selective cation channel Function: neuronal excitability, excitotoxicity, epileptogenesis and neurite outgrowth^33^ **Disorders**: none known	DN	Abs/Abs/Abs/Abs	MaxEnt: −100.0% NNSPLICE: −100.0% SSF: −100.0%	24.0/N.A.	N.A.	Likely pathogenic PS2 + PM2
*ERRFI1* NM_018948.4 c.566_567delCT; p.S189* Novel	Encodes: a cytoplasmic protein that is up‐regulated during cell‐growth. Function: regulation of neurite outgrowth and cortical neuron migration^34^ **Disorders**: none known	DN	Abs/Abs/Abs/Abs	N.A.	N.A./N.A.	N.A.	Likely pathogenic PS2 + PM2
*CTXN3* NM_001127385.2 c.164C>T; p.P55L Novel	Encodes: cortexin‐3. Function: may be involved in GABAergic neurotransmission during CNS development^35^ **Disorders**: none known	DN	Abs/Abs/Abs/Abs	Class C65 (GV: 0.00 ‐ GD: 97.78)	24.2/Del 0.00	HD: PD 1.00 HV: PD 0.999	Likely pathogenic PS2 + PM2 + PP3
*IRX6* NM_024335.3 c.1334C>A; p.A445E Novel	Encodes: a transcription factor. Function: within the CNS is not well studied. Possible role in neurogenesis^36^ **Disorders**: none known	DN	Abs/Abs/Abs/Abs	Class C15 (GV: 60.00 – GD: 76.03)	27.4/Del 0.01	HD: PD 1.00 HV: PD 0.992	Likely pathogenic PS2 + PM2 + PP3
*IQCA1* *NM_001270585.2* c.357 + 1G>T Novel	Encodes: ATPase Function: regulation of ciliary motility and the maintenance of the distal axoneme.^37^ A role in the CNS has not been established. **Disorders**: none known	DN	Abs/Abs/Abs/Abs	MaxEnt: −100.0% NNSPLICE: −100.0% SSF: −100.0%	N.A.	N.A.	Likely pathogenic (PS2, PM2)

Abs = Absent; ACMG = American College of Medical Genetics; AP = action potentials; AS = affected sibling; CNS = central nervous system; Del = deleterious; DD = developmental delay; DN = *De novo*; DS = Dravet syndrome; EIMFS = epilepsy of infancy with migrating focal seizures; FFEVF = familial focal epilepsy with variable foci; GATOR1 = Gap activity against Rags; GD = GnomAD; HD = HDivPred; HV:HVarPred; Inh = Inheritance; PD = Probably damaging; LTP = long‐term potentiation; MAF = minor allele frequency; NPLHS = neurodevelopmental disorder characterized by poor language and loss of hand skills PP = Polyphen; SeLECTs = Self‐limiting epilepsy with centrotemporal spikes; UF = unaffected father.

Deficits in components of the GATOR1 complex (NPRL2/NPRL3) result in increased mTORC1 signaling, causing increased levels of glycine that result in increased synaptic excitation via NMDARs and spontaneous seizures in mice^27^ (Fig 3B). Further investigation of the GABA_B_R2 p.R787C variant revealed that R787 lies within the well‐conserved intracellular coiled‐coil domain that mediates interactions with GABA_B_R1 (Fig 3C, D). Substitution of the basic amino acid, arginine with hydrophobic cysteine, is predicted to disrupt polar interactions within the hydrophobic core of this coiled‐coil domain (Fig 3E, F) and impair cell‐surface expression of GABA_B_R1/GABA_B_R2 heteromeric GABA_B_Rs.

Familial triome analysis identified 5 patients with *de novo* variants in candidate genes not previously reported in association with LKS or other types of epilepsy (see Table 4): *TRPC1* (c.961‐2A>C), *ERRFI1* (c.566_567delCT, [p.S189*]), *CTXN3* (c.164C>T, [p.P55L]), *IRX6* (c.1334C>A, [p.A445E]), and *IQCA1* (c.357 + 1G>T). Familial triome analysis using recessive and X‐linked recessive models of inheritance and CNV screening did not reveal any findings of significance.

## Discussion

Our study confirms that LKS confers significant morbidity. Akin to many DEEs – in addition to speech and language impairment and seizures – patients often have behavioral, cognitive, and motor difficulties. Despite this, some individuals can function independently in adulthood within a deaf/mute community. Early recognition of LKS is key to facilitate the necessary educational and social support package for affected individuals and their families.

Toso et al first suggested that early AOR might be associated with worse language outcome.^38^ Some cohort studies have corroborated this association,^5^, ^16^, ^21^, ^24^ whereas others have not.^17^, ^20^, ^22^ Both suggestions are plausible, reflecting contradictory perspectives: early vulnerability versus early plasticity.^39^ Review of 61 published LKS cases reported a negative correlation between AOR and outcome,^40^ although the variable methods used to rate language impairment is an acknowledged limitation. Our LKS study also found that, similar to other DEEs, there was a highly significant negative correlation between AOR and language outcome. To our knowledge, this is the first single center cohort study sufficiently powered for correlation analysis, with the advantage of standardized categorization of language impairment. Our finding supports the “early vulnerability” theory, proposing that an insult at a crucial stage of neurodevelopment is particularly damaging with “flow on” effects to the subsequent development of neural networks.

It is important to consider whether neuropsychological deficits are the result of epileptic processes, or whether they are part of a broader developmental process. The contribution of epileptiform activity is supported by the finding that the majority of patients have normal development prior to clinical presentation. Landau and Kleffner suggested in their landmark paper in 1957 that “*persistent convulsive discharge in brain tissue largely concerned with linguistic communication results in the functional ablation of these areas for normal linguistic behaviour*.”

ESES/CSWS has been strongly associated with neurocognitive impairment. The original definition of ESES/CSWS was a SWI of >85%,^6^ arbitrarily set, because this was the minimum SWI among the patients described.^6^ However, as it was subsequently proposed that neurocognitive deficits can occur at lower SWIs, the term “spike‐wave activation in sleep” (SWAS) was introduced.^3^, ^41^ In this paper, we have used the term “ESES/CSWS” specifically for SWI of >85% and “SWAS” for spike‐wave sleep activation in general. Due to the relapsing–remitting nature of LKS, longitudinal correlation of EEG findings to neuropsychological assessment findings is important. From our experience, EEG findings did not correlate well with language assessment scores. Some individuals regained language skills despite ongoing ESES/CSWS, and vice versa. In our dataset, the longitudinal effect of EEG as a predictor for EDQ was due to a significant difference between no sleep activation and >85% SWI. There was no significant difference between EEG‐0 and EEG‐1. EEG was also a significant predictor for behavioral difficulties. These results suggest that EEG abnormalities may, to an extent, be predictive of neuropsychological deficits in LKS. Indeed, the cutoff of 85% SWI for ESES/CSWS may actually have clinical significance, corroborating results from another recent study.^42^ Overall, EEG abnormalities are unlikely to be the only factor driving language and neuropsychological deficits, so management guided solely by EEG findings may lead to over‐treatment. This observation is worth emphasizing, because SWI reduction is commonly used as a treatment goal and measure of treatment outcome.^41^ We would suggest that treatment would be better tailored to clinical symptoms, rather than EEG findings.

Steroid therapy was a significant predictor of EDQ and RDQ scores. As in a recent multi‐center randomized controlled trial,^43^ this supports the use of steroid therapy in LKS with multidisciplinary input. Notably for EDQ and RDQ, the interaction between EEG and steroids is not significant, suggesting that although steroid therapy may alleviate neuropsychological impairment, it does so through unknown mechanisms distinct from just improving EEG.

Looking at genotype–phenotype, *GRIN2A‐*positive patients are more likely to have a medical history of speech and language delay and motor difficulties. In our cohort, all patients with *GRIN2A*‐positive LKS had some language recovery with treatment, but none improved to within normal range for age. This may reflect the effect of *GRIN2A* on neurodevelopment and supports findings from Viswanathan et al, regarding the distinction between DEE‐SWAS and EE‐SWAS.^44^ *GRIN2A* is also highly expressed in key areas of motor control (including the basal ganglia and cerebellum), which might explain motor findings. It is not clear why *GRIN2A‐*positive individuals seem to have fewer behavioral co‐morbidities.

The p.R518H in our cohort was previously reported^8^, ^45^ with functional investigations suggesting gain‐of‐function (GOF).^8^ Contrary to this, two subsequent studies found loss‐of‐function (LOF) effects.^46^, ^47^ Our study is also consistent with LOF, confirming reduced NMDAR surface expression and agonist potency. The p.R518C from our cohort affects the same amino acid with similar LOF, and even lower cell membrane expression and no detectable functional response. Detailed investigation of *GRIN2A* missense variants associated with EASD suggest both LOF and GOF functional effects^47^ (Supplementary Fig S1). However, for some variants (p.R518H and p.C436R), there are conflicting results. This underscores the importance of using different measures of NMDAR function (cell‐surface trafficking, agonist potency, and channel properties) before designing targeted therapeutic strategies.^47^

Variants associated with LKS result in LOF (see Supplementary Fig S1). This contradicts the theory that variants within the GluN2A transmembrane or linker domains are GOF with severe developmental phenotypes, whereas variants outside these domains are LOF with less severe developmental impairment.^48^ For LKS, most variants reported fall outside the transmembrane domains, result in LOF, and are associated with severe developmental impairment. Considering the role of NMDARs in excitatory neurotransmission and long‐term potentiation, it seems logical that GluN2A LOF variants lead to less prominent seizures but severe neurocognitive deficits.

New therapies are now emerging for disorders involving NMDARs. L‐serine, a precursor of the NMDAR co‐agonist, D‐serine, has been trialed in children with encephalopathy associated with LOF *GRIN2A/GRIN2B* mutations, and reported to improve seizure frequency, behavior, and neuro‐cognitive skills.^49^ Some LKS‐associated *GRIN2A* mutations, like those reported here, result in cellular mis‐localization; so pharmacological chaperones, such as Sigma‐1 receptor (S‐1R), which binds to and regulates GluN1 expression, may also have therapeutic potential. In murine models, S‐1R agonists have demonstrated rescue of neurocognitive defects caused by NMDAR antagonism. S‐1R modulation may well be considered as a future treatment strategy for LKS, having shown favorable neurocognitive benefits in schizophrenia.^50^

Our study has identified novel gene associations that, as for other DEEs, highlight the genetic heterogeneity and phenotypic pleiotropy in LKS (see Table 4). The variants identified in *GABBR2*, *SCN1A*, *NPRL3*, and *TRPC1* are most noteworthy. *ERRFI1*, *CTXN3*, *IRX6*, and *IQCA1* have not yet been reported in association with neurological disease. Although these genes are all highly expressed in the brain, their functions within the central nervous system are not well‐established.

A novel *de novo GABBR2* mutation was identified in a child with classical LKS, affecting R787, an evolutionarily well‐conserved amino‐acid within the coiled‐coil domain important for cell‐surface expression of GABA_B_R1^51^ (see Fig 3C–F). We also identified a *de novo SCN1A* mutation in a child with classical LKS. *SCN1A* mutations have been reported in 5 patients with EE‐SWAS phenotypes^31^, ^32^, ^44^ and p.V250I has been reported in 2 individuals with neurodevelopmental disorders/epilepsy.^25^, ^26^ The variant involves a highly conserved amino acid, located within S5 of the Na_v_1.1 first transmembrane domain, D1, an important pore‐lining region, where mutations cluster.

Additionally, this study identified *NPRL3* variants in 2 LKS families. c.767 + 1G>T was identified in 2 siblings (one with LKS) with a strong family history of epilepsy, predicted to cause a splicing defect and exon 7 skipping. It is also reported on ClinVar (RCV000703657.1) for a proband with familial focal epilepsy with variable foci. The other *NPRL3* variant, p.P322L, has not been previously reported and is a variant of uncertain significance, although absent from control population databases and predicted deleterious by most *in silico* algorithms. P322 is a highly‐conserved amino acid located within the intermediary domain, important for interaction with NPRL2. Interestingly, a pathogenic, maternally inherited variant in *NPRL2* has been reported in another individual with EE‐SWAS and polymicrogyria.^44^ Both *NPRL3* variants identified in this cohort were inherited from unaffected parents. Notably, incomplete penetrance has been well‐documented for GATOR1 complex mutations, whereas regulation of mTOR activity may be influenced by environmental factors.^27^

Finally, a splice‐site variant was identified in *TRPC1*, predicted to cause exon 7 skipping. *TRPC1* mutations have not previously been reported in epilepsy, despite associations between TRPC channel function, epileptogenesis, and memory/learning.^33^ This may be the first report of a *TRPC1* variant associated with epilepsy. Functional investigations and interrogation of other patient cohorts will help clarify the link between *TRPC1* and LKS.

*GRIN2A* is a fitting gene for a DEE like LKS because, encoding a subunit of the NMDAR, it plays a key role in long‐term potentiation (LTP), an important process for learning and memory formation. Interestingly, *GABBR2*, *SCN1A*, *NPRL3*, and *TRPC1* all have links to NMDAR function/LTP. Physiologically, NMDAR activation increases *GABBR1* expression. Cell‐surface expression of GABA_B_R1 is likely blocked by *GABBR2* p.R787C. It has also been demonstrated that enhancement of GluN2A activity ameliorates seizure activity and cognitive deficits in *SCN1A* knock‐in mice.^52^ As mentioned above, NPRL2/NPRL3 deficits are linked to increased NMDAR activation. Last, co‐activation of NMDAR and TRPC channels is required for chemical LTP, as the latter helps facilitate calcium influx.^33^ These relationships bring to light the central role of LTP mechanisms in LKS.

This study represents the largest LKS cohort study to date, allowing us to delineate key clinical features, co‐morbidities, electrophysiology, treatment efficacy, clinical outcomes, and prognostic factors. We have identified pathogenic *GRIN2A* variants in 15.5% of the cohort, and report detailed functional characterization of LOF mutations in a critical agonist‐binding region of GluN2A, including analysis in artificial synapses. Last, new potentially pathogenic variants in *GABBR2*, *SCN1A*, *TRPC1*, *ERRFI1*, *CTXN3*, *IRX6*, and *IQCA1* have been identified in *GRIN2A‐*negative individuals, emphasizing the underlying genetic heterogeneity of LKS. Taken together with a recent study on the molecular landscape of DEE‐SWAS that identified pathogenic variants in other novel genes,^44^ it is clear that an in‐depth understanding of the pathophysiology and genetics of DEE syndromes will facilitate development of better precision therapies and improve patient outcomes.

## Author Contributions

**Adeline Ngoh:** Conceptualization; data curation; funding acquisition; investigation; methodology; project administration; resources; writing – original draft; writing – review and editing. **Maria Clark:** Data curation; formal analysis. **Rebecca Greenaway:** Formal analysis; investigation. **Xiumin Chen:** Formal analysis; investigation. **Kimberley M. Reid:** Formal analysis; investigation. **Katy Barwick:** Formal analysis; investigation. **Esther Meyer:** Formal analysis; investigation. **Dale Moulding:** Formal analysis; investigation. **Natalie Trump:** Formal analysis; investigation. **J. Helen Cross:** Formal analysis; investigation. **Sean D. Fraser:** Formal analysis; investigation. **Lachlan de Hayr:** Formal analysis; investigation. **Dimitri M. Kullmann:** Formal analysis; investigation. **Joseph W. Lynch:** Formal analysis; investigation. **Robert J. Harvey:** Formal analysis; investigation; supervision; writing – review and editing. **Manju A. Kurian:** Data curation; formal analysis; investigation; methodology; project administration; resources; supervision; writing – review and editing.

## Potential Conflicts of Interest

The authors have no conflicts of interest to declare.

## Supporting information


**Data S1.** Supporting Information.

## Data Availability

De‐identified data collected and presented in this study, including individual participant data, can be made available upon reasonable request after publication of this article. Data can be requested by contacting the corresponding author.
