# Supplementary material for: Clinical and Molecular Genetic Characterization of Landau Kleffner Syndrome: An Observational Cohort and Experimental Study
Source: Ann Neurol. 2025 Sep 13;98(5):951–66. doi: 10.1002/ana.27306 (PMC12577679; doi:10.1002/ana.27306)
Supplement: Supplementary file 1 — Data S1. Supporting Information. [file ANA-98-951-s001.docx]

**Supplementary information**

**Clinical and Molecular Genetic Characterization of Landau Kleffner Syndrome: An Observational Cohort and Experimental Study**

Adeline Ngoh PhD^1,2^, Maria Clark MRCP^3^, Rebecca Greenaway PhD^4^, Xiumin Chen PhD^5^, Kimberley M. Reid PhD^1^, Katy Barwick PhD^1^, Esther Meyer PhD ^1^, Dale Moulding PhD ^6^, Natalie Trump PhD ^1^, J Helen Cross PhD ^1,3^, Sean D. Fraser^7,8^, Lachlan de Hayr PhD ^7,8^, Dimitri M. Kullmann PhD^9^, Joseph W. Lynch PhD ^5^, Robert J. Harvey PhD ^7,8^, Manju A. Kurian PhD ^1,3^

**Running title**: Landau Kleffner Syndrome

**Affiliations**

1. Developmental Neurosciences, UCL Great Ormond Street Institute of Child Health, Zayed Centre for Research into Rare Disease in Children, London, UK
2. Paediatric Neurology, KK Women’s and Children’s Hospital, Singapore
3. Department of Neurology, Great Ormond Street Hospital, London, UK
4. Department of Psychology, Great Ormond Street Hospital, London, UK
5. Queensland Brain Institute, The University of Queensland, Brisbane, QLD Australia
6. Developmental Biology and Cancer Programme, UCL Great Ormond Street Institute of Child Health, London, UK.
7. School of Health, University of the Sunshine Coast, Queensland, Australia
8. National PTSD Research Centre, Thompson Institute, Birtinya, Queensland, Australia
9. UCL Queen Square Institute of Neurology, London UK

Supplementary Methods 3

Patient Assessment 3

Molecular genetic analysis 3

Gene expression, protein expression and protein localization 3

Electrophysiology 5

Supplementary Tables 6

Supplementary Table 1: Classification of Language outcomes 6

Supplementary Table 2: Classification of outcomes as adults 6

Supplementary Table 3: List of genes associated with epilepsy and epilepsy aphasia spectrum disorders 7

Supplementary Table 4: Characteristics of patients with no sleep EEG record available 8

Supplementary Table 5: Differences in collected variables among language outcome groups 8

Supplementary Table 6: Test of model effects, parameter estimates and pairwise comparisons 9

Supplementary Table 7: Phenotypic comparison between *GRIN2A*-positive (n=7) and *GRIN2A*-negative (n=38) individuals in study cohort 11

Supplementary Table 8: Clinical features for *GRIN2A*-positive LKS individuals reported in the literature 11

Supplementary Table 9: Clinical features for *GRIN2A*-negative LKS individuals reported in the literature 12

Supplementary Table 10: Phenotypic comparison between *GRIN2A*-positive (n=22) and *GRIN2A*-negative individuals (n=53) – Study data combined with data from literature reports 12

Supplementary Figure 1 13

Supplementary References 14

# Supplementary Methods

## Patient Assessment

*Developmental Assessments*: Due to the nature of LKS and the longitudinal aspect of developmental assessments, a variety of assessments were used. Where possible, Clinical Evaluation of Language Fundamentals (CELF) was used to obtain expressive and receptive language scores and Griffiths, Wechsler Preschool and Primary Scale of Intelligence (WPPSI), and/or Wechsler Intelligence Scale for Children (WISC) assessments were used to gain a non-verbal intelligence quotient (IQ). However, other developmental assessments were used when these instruments were not appropriate for the child’s developmental or language level. As the assessments have been conducted over a course of more than 20 years, different versions of these assessments were used. Most children received a combination of more than one method of testing during the course of their illness. In cases where a child demonstrated particular attentional or behavioural difficulties, and professionals were unable to conduct full, formal assessment using the age appropriate assessment, a developmental quotient (DQ) score was given.

*Developmental Quotient*: Developmental Quotients were calculated as (developmental age equivalent / chronological age) × 100.

## Molecular genetic analysis

For Sanger sequencing, the gene structure of *GRIN2A* (ENSG00000183454) was obtained from Ensembl genome browser (http://www.ensembl.org/index.html). Primer pairs were designed based on all Ensembl coding transcript variants using Primer3 software (http://bioinfo.ut.ee/primer3/) and designs are available on request. Multiplex Ligation Probe Amplification (MLPA) was carried out to look for *GRIN2A* copy number variants using an MLPA kit (SALSA MLPA P410 *GRIN2A*/*GRIN2B* probemix and SALSA MLPA EK1 reagent kit, MRC Holland) according to manufacturer’s instructions.

The clinical epilepsy gene panel test at North East Thames Regional Genetics Laboratory screens 72 genes associated with severe delay and seizures using next-generation sequencing (Agilent Sure-Select + MiSeq). Variants are confirmed with Sanger Sequencing.

For whole-exome sequencing and whole-genome sequencing, 2μg of genomic DNA (50ng/μl) sample was sent to Beijing Genomics Institute (BGI) - Hong Kong. Library construction was performed there. Samples from each triome were sequenced per lane to an overall coverage of 100× using the Illumina HiSeq 2500 sequencing platform. Raw data was returned in.fastq file format (2× per sample). First- pass quality control analysis was performed using our established bioinformatic pipeline, including mapping of reads to the reference genome, calling and recalibration of variant calls, and the production of a list of high-quality variants.^1^ Variant call files (.vcf) were uploaded into the Qiagen Ingenuity Variant analysis (QIVA) software platform. A set of pre-determined filters were used to remove low confidence variants (call quality < 20, read-depth <10, allele fraction < 30), common variants (allele frequency > 0.1% according to 1000 Genomes Project, Exome Aggregation Consortium, Genome Aggregation Database, or Exome Server Project), and likely benign variants. Each set of triome data was then analysed separately for variants with *de novo*, recessive and X-linked recessive models of inheritance. To identify variants within a panel of known epilepsy genes and genes previously associated with EASD, a list of specified genes (Supplementary Table 3) was uploaded onto the QIVA platform and the program was asked to filter for variants within the genes of interest.

## Gene expression, protein expression and protein localization

#### **Site-directed mutagenesis**

We carried out site-directed mutagenesis on plasmid expression construct pRK5-GluN2A^1^ to generate pRK5-GluN2A^R518C^ and pRK5-GluN2A^R518H^ constructs using the Quikchange Lightning Site-Directed Mutagenesis Kit (Agilent Technologies) according to the manufacturer’s protocol. Primer pair sequences are available on request. After site-directed mutagenesis, mutant plasmids were validated with Sanger DNA sequencing.

#### **Cell culture**

HEK293 cells were cultured in 25cm^2^ Corning tissue culture flasks (ThermoFisher) containing 5ml of Dulbecco’s Modified Eagle’s Medium (DMEM) supplemented with 10% (v/v) fetal bovine serum (FBS), and 10,000 units/ml penicillin/streptomycin. The cells were incubated in a 5% CO_2_ incubator at 37°C and passaged twice a week. When the HEK293 cells were 80-90% confluent in the 25cm^2^ flask, they were plated onto either Corning 6-well polystyrene plates (ThermoFisher) for reverse transcription polymerase chain reaction (RT-PCR) and Western blotting experiments or 4-well Lab-tek chamber slides for immunocytochemistry experiments.

#### **Transfection**

12 to 24 hours after plating, HEK293 cells were co-transfected with plasmids pRK5-GluN1^2^ together with either wild-type pRK5-GluN2A, pRK5-GluN2A^R518C^ or pRK5-GluN2A^R518H^ in a 1:1 ratio, using Lipofectamine-2000 (ThermoFisher) according to manufacturer’s instructions. 8µg of wild-type pRK5-GluN1 plasmid DNA and 8µg of pRK5-GluN2A, pRK5-GluN2A^R518C^ or pRK5-GluN2A^R518H^ plasmids were used for transfection in each well of the Corning 6-well plates, and 2µg of each plasmid DNA was used for transfection in each well of the Lab-tek chamber slides. 16µl of the NMDA receptor antagonist, D-2-amino-5-phosphonopentanoic acid (D-AP5) (200µmol/l) was added to each well of the 6 well plate, and 4µl of D-AP5 (200µmol/l) was added to each well of the Lab-tek chamber slide to protect the cells from NMDAR-mediated cytotoxicity.

#### **Reverse Transcription polymerase chain reaction (RT-PCR)**

RT-PCR was performed on cell lysates obtained from HEK293 cells transfected with plasmids encoding wild-type GluN1 and either wild-type or mutant GluN2A to compare wild-type and mutant GluN2A mRNA levels. Total ribonucleic acid (RNA) was extracted from cell pellets collected after 24 hours of transfection. This procedure was performed using an RNeasy Mini Kit (Qiagen) using the manufacturer’s instructions. Purification and reverse transcription of RNA was carried out using the QuantiTect Reverse Transcription Kit (Qiagen). Each reaction was carried out in triplicate on a skirted 96-well plate. Primer sequences are available on request. Reactions were run on the Applied Biosystems StepOne Real-Time PCR System (ThermoFisher). Relative mRNA levels were calculated for each GluN2A construct in relation to wild-type using the delta-delta CT (ΔΔCT) method. ^3^ Mean and standard deviation of relative expression values were obtained through triplicate measurements for each genotype. One-way ANOVA was performed in Prism 7.0 (Graph Pad) to compare the difference in mRNA levels between wild-type GluN2A transfected and GluN2A R518C/R518H transfected cells.

#### **Western blotting**

24 hours after transfection, cell-lysate protein samples (10µg/well) were prepared in 4× Laemmli buffer (Bio-Rad) and 5× Dithiothreitol (DTT) (ThermoFisher). The samples were then heated at 37°C for 15 minutes and spun down briefly before loading onto appropriate wells on 4-20% Mini-Protean TGX Stain-Free Pre-cast gels (Bio-Rad). Gels were ran at 200V for 50 minutes. Proteins were then transferred onto a polyvinylidene difluoride (PVDF) membrane from the Trans-blot Turbo Mini PVDF transfer pack (Bio-Rad), using the Trans-Blot Transfer System. After protein transfer, the PVDF membrane was cut at the 50kDa marker. The top half (>50kDa, Part A) was blocked then incubated at 4°C overnight with 1:1,000 GluN2A antibody (M264, Sigma-Aldrich). The bottom half (<50kDa, Part B) was blocked then incubated at 4°C overnight with 1:5,000 GAPDH antibody conjugated with horse radish peroxidase (HRP) (Cell Signalling Technology), in PBS with 0.5% Tween-20. Protein bands were imaged using the ChemiDoc™ MP Imaging System (Biorad). Results were analysed using ImageJ (https://imagej.nih.gov/ij/). The density of each protein band was measured using ImageJ and normalized to GAPDH. The result for each mutation was then compared to that of wild-type GluN2A to obtain relative protein expression for GluN2A^R518C^ and GluN2A^R518H^. Mean and standard deviation values were obtained through measurements from three independent transfections. One-way ANOVA was performed in Prism 7.0 (Graph Pad) to compare the difference in protein expression between each mutation and wild-type N2A transfected cells.

#### **Immunocytochemistry**

24 hours after transfection, cell culture medium was aspirated and replaced with 1% paraformaldehyde (PFA) in complete media. The cells were then incubated at room temperature for 20 minutes and rinsed with 1× phosphate buffered saline (PBS), before they were fixed by incubation with 4% PFA in 1× PBS at room temperature. The cells were incubated in blocking buffer for 30 minutes at room temperature then incubated overnight at 4°C with 1:1000 anti-GluN2A extracellular antibody (Alomone Labs). The following day, the cells were washed thrice in 1× PBS then incubated for 45 minutes at room temperature with 1:800 Alexa-Fluor Goat Anti-rabbit 594nm IgG secondary antibody (Abcam) and 1:1000 CF™488A wheat germ agglutinin (Biotium) in 1× PBS with Ca^2+^ and 10% FBS. Nuclei were counterstained with 1:1000 4,6-diamidino-2-phenylindole (DAPI) (Sigma-Aldrich). Images were captured on the Zeiss LSM 710 inverted confocal microscope and analysed using Fiji software (https://fiji.sc/). A macro for Fiji software was designed to automatically measure the intensity of GluN2A immunofluorescence co-localizing with the membrane surface marker, wheat germ agglutinin (WGA) and to count the number of nuclei counted in each image. The intensity of GluN2A immunofluorescence co-localizing with WGA was averaged to the number of nuclei counted. The results obtained for GluN2A ^R518C^/GluN2A^R518H^ transfected cells were compared to the results obtained for wild-type GluN2A transfected cells to obtain relative surface-expression values. Mean and standard deviation of these relative expression values were obtained through repeating measurements for three independent transfections. One-way ANOVA was performed in Prism 7.0 (Graph Pad) to compare the difference in surface expression between GluN2A^R518C^/GluN2A^R518H^ transfected and wild-type GluN2A transfected cells.

## Electrophysiology

Neurons for artificial synapse recordings were prepared as previously described.^4^ Artificial synapse recordings were made in the whole-cell patch-clamp recording configuration at a holding potential of −70 mV. All electrophysiological experiments were performed at room temperature (22 ± 2°C). Patch pipettes were fabricated from borosilicate hematocrit tubing (Harvard Apparatus, Germany) with tip resistances of 2-5 MΩ when filled with the intracellular solution which contained (in mM): 145 CsCl, 2 CaCl_2_, 2 MgCl_2_, 10 HEPES, and 10 EGTA, adjusted to pH 7.4 with CsOH. Cells were perfused with extracellular solution, which contained (in mM): 140 NaCl, 5 KCl, 2 CaCl_2_, 10 HEPES and 10 D-glucose, adjusted to pH 7.4 with NaOH. Synaptic currents were filtered (−3dB, 4-pole Bessel) at 4 kHz and sampled at 10 kHz and recorded using a Multiclamp 700B amplifier and pClamp 10 software (Molecular Devices, Sunnyvale, CA, USA). Recordings with series resistances >20 MΩ were discarded and series resistance compensation was not applied to the recorded cell. Solutions containing defined concentrations of glutamate and glycine were applied to cells via gravity-induced perfusion via parallel microtubules. All the chemicals employed for this part of the study were obtained from Sigma-Aldrich.

# Supplementary Tables

## Supplementary Table 1: Classification of Language outcomes

| **Classification** | **Percentile** | **Standard deviation (SD)** | **Age equivalence** |
| --- | --- | --- | --- |
| Average/Normal language | > 25th centile | Within 1 SD | Age equivalent |
| Mild impairment | 12.5th centile to 25th centile | Between -1 to - 1.5 SD | >75% of chronological age |
| Moderate impairment | 1st centile to 12.5th centile | Between - 1.5 to 2 SD | 50% to 75% of chronological age |
| Severe impairment | < 1st centile | < 2 SD or unable to use age appropriate scale | < 50% chronological age |
| No functional speech | Unable to verbalize or difficult to decipher single words |  |  |

As results obtained from different assessments were not always truly comparable, where possible, percentile measures were obtained for each respective test, based on the average score expected for the child’s age, as an approximate means for comparison. Where only age-equivalent results were available, age equivalence of half the chronological age (DQ of 50) or less was taken to define severe language impairment as suggested by previous literature. ^5^ Age equivalence of more than half but less than 75% of the chronological age was arbitrarily defined as moderate language impairment. Where more than one assessment was used during the same clinic attendance and a range of scores was obtained, e.g. receptive language 25^th^ centile, and expressive language 10^th^ centile, the worse/worst score (10^th^ centile) was recorded.

## Supplementary Table 2: Classification of outcomes as adults

| **Classification** | **Definition** |
| --- | --- |
| Independent with mild or no language difficulty | Not dependent on a caregiver (in employment or studying in a mainstream institution), and who have no/minimal difficulty with day to day conversations |
| Independent with significant language difficulty | Not dependent on a caregiver, but whose language difficulties remain a disability – e.g. those relying on sign language, or those educated within special schools |
| Dependent | Reliant on a care-giver (parental or institutional care) |

## Supplementary Table 3: List of genes associated with epilepsy and epilepsy aphasia spectrum disorders

| *AARS* | *C3orf58* | *CSTB* | *GABRA1* | *ITPA* | *MBD5* | *PCDH19* | *RNMT* | *SLC9A9* | *TTC27* |
| --- | --- | --- | --- | --- | --- | --- | --- | --- | --- |
| *AATF* | *C7orf55* | *CTNNA2* | *GABRB1* | *KAT8* | *MDGA2* | *PCYT1A* | *RPL38* | *SLITRK2* | *TTYH2* |
| *ACACA* | *CACNA1A* | *CTNNA3* | *GABRB3* | *KCNA1* | *MDH2* | *PDE4D* | *RRN3* | *SMAD3* | *TXNIP* |
| *ADAM22* | *CACNA1D* | *CYFIP2* | *GABRD* | *KCNA2* | *MECP2* | *PDXDC1* | *RYR3* | *SMC1A* | *UBA5* |
| *ADGRV1* | *CACNA2D1* | *DDX52* | *GABRG2* | *KCNB1* | *MEF2C* | *PEG10* | *SAMD11* | *SNX16* | *UBE2A* |
| *AJAP1* | *CACNB4* | *DENND5A* | *GBE1* | *KCNC1* | *METTL7A* | *PHF8* | *SAMD4A* | *SOCS4* | *UBE3A* |
| *ALG13* | *CAD* | *DEPDC5* | *GCH1* | *KCNIP4* | *MGC16275* | *PIGA* | *SCARB2* | *SPTAN1* | *UBXN7* |
| *ANKRD30B* | *CASP2* | *DHRS11* | *GGNBP2* | *KCNJ10* | *MPDZ* | *PIGP* | *SCN1A* | *SRPX2* | *UNC13C* |
| *AP3B2* | *CBLN1* | *DIAPH3* | *GIPC1* | *KCNMA1* | *MRM1* | *PIGW* | *SCN1B* | *ST3GAL3* | *VKORC1* |
| *ARFGEF1* | *CCL2* | *DIP2B* | *GMFB* | *KCNQ2* | *MSC* | *PLCB1* | *SCN2A* | *STS* | *WDHD1* |
| *ARFGEF2* | *CD300A* | *DLG2* | *GNAO1* | *KCNQ3* | *MSTO1* | *PLK2* | *SCN3A* | *STX1B* | *WDR45* |
| *ARHGEF15* | *CD59* | *DNM1* | *GOSR2* | *KCNT1* | *MSTO2P* | *PLOD2* | *SCN8A* | *STX4* | *WDR45B* |
| *ARHGEF4* | *CDH13* | *DOCK7* | *GPAA1* | *KCNT2* | *MUC20* | *PLPBP* | *SCN9A* | *STXBP1* | *WWOX* |
| *ARHGEF9* | *CDH9* | *DUSP14* | *GPHN* | *KCTD7* | *NACC1* | *PNKP* | *SDHAP1* | *SYNGAP1* | *YWHAG* |
| *ARV1* | *CDKL5* | *DYRK1A* | *GPR37L* | *KCTD7* | *NBPF11* | *PNPLA4* | *SETD5* | *SYNJ1* | *YY1AP1* |
| *ARX* | *CDKN3* | *EEF1A2* | *GPRC5C* | *KIAA0831* | *NCOA2* | *POLR3GL* | *SHANK3* | *SYNRG* | *ZFAND1* |
| *ASH1L* | *CGRRF1* | *EFHC1* | *GRID2* | *KIF19* | *NECAP1* | *PRICKLE1* | *SIK1* | *SZT2* | *ZNF519* |
| *ASIC2* | *CHD2* | *EIF3E* | *GRIN1* | *KLH17* | *NEXMIF* | *PRICKLE2* | *SLC10A5* | *TADA2A* | *ZNF646* |
| *ATF1* | *CHRNA2* | *ELP4* | *GRIN2B* | *KTN1-AS1* | *NHLRC1* | *PRRT2* | *SLC11A2* | *TBC1D24* | *ZNF668* |
| *ATP1A3* | *CHRNA4* | *EPHB2* | *GRIN2D* | *L2HGDH* | *NID2* | *PRSS53* | *SLC12A5* | *TBL1XR1* | *ZNHIT3* |
| *ATP6AP2* | *CHRNB2* | *EPM2A* | *GRIP1* | *LACTB2* | *NOC2L* | *PTGER3* | *SLC13A5* | *TCF4* |  |
| *ATP6V1A* | *CLTC* | *ERBB4* | *GUF1* | *LARP4* | *NPRL3* | *PTPRT* | *SLC1A2* | *TCTEX1D2* |  |
| *ATRX* | *CNIH* | *FBXO34* | *HCN1* | *LETMD1* | *NRG3* | *PURA* | *SLC1A4* | *TFCP2* |  |
| *BCDK* | *CNPY3* | *FBXO8* | *HDHD1* | *LGALS3* | *NRXN1* | *QARS* | *SLC25A12* | *TFRC* |  |
| *BIRC6* | *CNTNAP2* | *FGD2* | *HNRNPU* | *LGI1* | *NTAN1* | *RAB3C* | *SLC25A22* | *TMEM132D* |  |
| *BRAT1* | *CNTNAP2* | *FHF1* | *HSBP1* | *LHX1* | *NTRK2* | *RBFOX1* | *SLC2A1* | *TMEM132E* |  |
| *BRINP3* | *COL18A1* | *FOXG1* | *HSPG2* | *LRRTM4* | *PACS2* | *RBFOX3* | *SLC30A3* | *TMEM139* |  |
| *BSN* | *CPLX1* | *FOXP2* | *IMPA1* | *LTBP1* | *PAX6* | *REG1A* | *SLC35A2* | *TMEM257* |  |
| *BTBD17* | *CSPP1* | *FRRS1L* | *IQSEC2* | *MAPK10* | *PCDH12* | *RELN* | *SLC6A1* | *TNK2* |  |
| *C17orf102* | *CSRNP2* | *GABBR2* | *ITGB1BP1* | *MAPK1IP1L* | *PCDH15* | *RHOBTB2* | *SLC7A6OS* | *TRAM1* |  |

## Supplementary Table 4: Characteristics of patients with no sleep EEG record available

| **Patient** | **1** | **2** | **3** | **4** | **5** | **6** | **7** | **8** | **9** |
| --- | --- | --- | --- | --- | --- | --- | --- | --- | --- |
| **Male** | Yes | No | No | Yes | No | Yes | No | Yes | Yes |
| **Follow up (months)** | 297 | 298 | 281 | 313 | 302 | 47 | 307 | 327 | 294 |
| **+ve FH** | Yes | Yes | No | Yes | No | No | No | Yes | No |
| **SD** | Yes | No | No | Yes | No | No | No | No | No |
| **SLR age** | 8y10m | 3y6m | 5y | 5y9m | 5y6m | 3y10m | 5y5m | 7y7m | 5y2m |
| **Sz** | Yes | Yes | No | Yes | Yes | Yes | Yes | Yes | Yes |
| **EEG findings** | Very frequent SWs in B/L CT regions | Frequent SWs in B/L posterior temporal regions R>L | Frequent SWs in L parietal and posterior temporal regions | Frequent SWs in mid and posterior temporal regions L> R | B/L temporal lobe SWs | Frequent SWs in B/L CT regions  SA (Stage 1 sleep only) | B/L temporal SWs L>R | B/L temporal SWs | SWs over posterior regions R>L. Marked increase on eye closure. Did not fall asleep |
| **< ave NVS** | Yes | Yes | Yes | Yes | No | No | No | Yes | No |
| **BD** | Yes | Yes | Yes | Yes | Yes | Yes | Yes | Yes | No |
| **Lang. OC at last FU** | MDI | SI | SI | NS | NL | NL | NL | SI | NL |
| **Sz at last FU** | Yes | No | No | No | No | No | No | Yes | No |
| **OC at >18y** | NL-MI/I | MDI-SI/I | MDI-SI/I | MDI-SI/I | NL-MI/I | N.A. | NL-MI/I | MDI-SI/I | NL-MI/I |
| ***GRIN2A* mutation** | c.1552C>T  p.Arg518Cys | N.A. | c.2041C>Tp.Arg681* | N.A. | N.A. | N.A. | N.A. | N.A. | N.A. |

ave: average; BD: behavioural disorder; B/L: bilateral; +ve FH: family history of seizures or speech and language impairment; FU: follow-up; m: months; I: independent; L: Left; Lang.: language; MI: mild speech impairment; MDI: moderate speech impairment; N.: number of patients; NL: normal language; NS: no speech; NVS: non-verbal skills; OC: outcome; R: right; SA: sleep activation; SD: pre-existing speech delay; SI: severe speech impairment; SLR: speech and language regression; SW: spike-waves; Sz: clinical seizures; y: years

## Supplementary Table 5: Differences in collected variables among language outcome groups

|  | **Normal language** | **Mild impairment** | **Moderate impairment** | **Severe Impairment** | **No functional**  **language** | **(*p* value)** |
| --- | --- | --- | --- | --- | --- | --- |
| **N:** | 14 (26.9%) | 8 (15.4%) | 10 (19.2%) | 12 (23.1%) | 8 (15.4%) |  |
| **Mean current age (range)** | 18y8m ± 8y9m  (7y6m to 31y) ^§^ | 18y4m ± 6y2m  (10y10m to 28y1m) ^§^ | 17y0m ± 7y0m  (7y3m to 28y2m) ^§^ | 16y9m ± 8y0m  (9y6m to 30y3m) ^§^ | 21y9m ± 9y4m  (9y8m to 33y9m) ^§^ | 0.689* |
| **Male%** | 50.0% | 62.5% | 90.0% | 50.0% | 50.0% | 0.270^†^ |
| **Positive FH** | 21.4% | 37.5% | 60.0% | 50.0% | 50.0% | 0.362^†^ |
| **Mean age at SLR (range)** | 5y6m ± 1y6m  (3y3m to 8y0m)^§^ | 6y6m ± 2y3m  (4y8m to 11y10m) ^§^ | 4y1m ± 2y5m  (1y6m to 8y10m) ^§^ | 4y7m ± 1y6m  (2y6m to 7y7m)^§^ | 3y2m ± 1y4m  (1y6m to 4y9m)^§^ | **0.006*** |
| **Prior speech and language delay** | 21.4% | 37.5% | 50.0% | 25.0% | 12.5% | 0.409^†^ |
| **Clinical Sz** | 92.9% | 75.0% | 80.0% | 75.0% | 100.0% | 0.438^†^ |
| **ESES on EEG (No.)** ^‡^ | 80.0%  (8/10) | 62.5%  (5/8) | 66.7%  (6/9) | 66.7%  (6/9) | 57.1%  (4/7) | 0.886^†^ |
| **< average NVIQ** | 35.7% | 25.0% | 40.0% | 83.3% | 37.5% | 0.058^†^ |
| **BD** | 71.4% | 62.5% | 60.0% | 100.0% | 87.5% | 0.142^†^ |
| **MD** | 21.4% | 25.0% | 40.0% | 41.7% | 12.5% | 0.551^†^ |
| **Ongoing Sz** | 21.4% | 12.5% | 30.0% | 25.0% | 12.5% | 0.880^†^ |
| **Ongoing ESES**^‡^ | 10.0% | 0.0% | 11.1% | 22.2% | 28.6% | 0.517^†^ |

BD: behavioural difficulties; MD: motor difficulties; ESES: electrical status epilepticus in slow wave sleep; FH= family history; N= number of patients; NVIQ: non-verbal intelligence quotient; SLR: speech and language regression; Sz: seizure. *One-way Anova with Bonferroni correction; ^†^Fisher’s exact; ^‡^those without available sleep EEG reports (9/52, 17.3%) were excluded; ^§^standard deviation

## Supplementary Table 6: Test of model effects, parameter estimates and pairwise comparisons

**A: Test of Model effects**

| **Dependent Variable:** | **Expressive DQ** | | | **Receptive DQ** | | | **Non-verbal DQ** | | | **Extreme Behaviour** | | |
| --- | --- | --- | --- | --- | --- | --- | --- | --- | --- | --- | --- | --- |
|  | **Wald Chi-Square** | **Df** | **Sig** | **Wald Chi-Square** | **Df** | **Sig** | **Wald Chi-Square** | **Df** | **Sig** | **Wald Chi-Square** | **Df** | **Sig** |
| **(Intercept)** | 336.848 | 1 | 0.000 | 428.923 | 1 | 0.000 | 6490.608 | 1 | 0.000 | 1.631 | 1 | 0.202 |
| **Age at Ax** | 8.959 | 1 | 0.003 | 7.337 | 1 | 0.007 | 4.278 | 1 | 0.039 | 2.735 | 1 | 0.098 |
| **Steroids** | 11.541 | 1 | 0.001 | 7.573 | 1 | 0.006 | 0.001 | 1 | 0.975 | 1.582 | 1 | 0.208 |
| **EEG** | 6.059 | 2 | 0.048 | 4.692 | 2 | 0.096 | 5.087 | 2 | 0.079 | 7.569 | 2 | 0.023 |

Ax: Assessment, EEG: electroencephalogram; Sig: significance

**B: Parameter Estimates**

| **Dependent variable** | **Expressive DQ** | | | | | | | **Receptive DQ** | | | | | | |
| --- | --- | --- | --- | --- | --- | --- | --- | --- | --- | --- | --- | --- | --- | --- |
| Parameter | B | Std. Error | 95% Wald Confidence Interval | | Hypothesis Test | | | B | Std. Error | 95% Wald Confidence Interval | | Hypothesis Test | | |
|  |  |  | Lower | Upper | Wald Chi-Square | df | Sig |  |  | Lower | Upper | Wald Chi-Square | df | Sig |
| (Intercept) | 3.214 | 0.2295 | 2.764 | 3.664 | 196.151 | 1 | 0.000 | 3.424 | 0.2113 | 3.010 | 3.838 | 262.600 | 1 | 0.000 |
| ageatAx | 0.062 | 0.0208 | 0.022 | 0.103 | 8.959 | 1 | **0.003** | 0.052 | 0.0194 | 0.014 | 0.090 | 7.337 | 1 | **0.007** |
| [steroids=1] | 0.321 | 0.0946 | 0.136 | 0.507 | 11.541 | 1 | **0.001** | 0.282 | 0.1023 | 0.081 | 0.482 | 7.573 | 1 | **0.006** |
| [steroids=0] | 0^a^ | . |  |  |  |  |  | 0^a^ |  |  |  |  |  |  |
| [EEG=2] | -0.288 | 0.1421 | -0.567 | -0.010 | 4.119 | 1 | **0.042** | -0.282 | 0.1304 | -0.538 | -0.027 | 4.686 | 1 | **0.030** |
| [EEG=1] | -0.014 | 0.1134 | -0.237 | 0.208 | .016 | 1 | 0.899 | -0.106 | 0.1212 | -0.343 | 0.132 | 0.760 | 1 | 0.383 |
| [EEG=0] | 0^a^ | . | . | . | . | . | . | 0^a^ |  |  |  |  |  |  |
| (Scale) | 0.351 |  |  |  |  |  |  | 0.380 |  |  |  |  |  |  |

| **Dependent variable** | **Non-verbal DQ** | | | | | | | **Extreme Behaviour** | | | | | | |
| --- | --- | --- | --- | --- | --- | --- | --- | --- | --- | --- | --- | --- | --- | --- |
| Parameter | B | Std. Error | 95% Wald Confidence Interval | | Hypothesis Test | | | B | Std. Error | 95% Wald Confidence Interval | | Hypothesis Test | | |
|  |  |  | Lower | Upper | Wald Chi-Square | df | Sig |  |  | Lower | Upper | Wald Chi-Square | df | Sig |
| (Intercept) | 4.564 | 0.0667 | 4.434 | 4.695 | 4689.223 | 1 | 0.000 | 0.514 | 0.9198 | -1.289 | 2.316 | 0.312 | 1 | 0.577 |
| ageatAx | -0.015 | 0.0073 | -0.029 | -0.001 | 4.278 | 1 | **0.039** | -0.168 | 0.1017 | -0.367 | 0.031 | 2.735 | 1 | 0.098 |
| [steroids=1] | 0.001 | 0.0300 | -0.058 | 0.060 | 0.001 | 1 | 0.975 | -0.389 | 0.3094 | -0.996 | 0.217 | 1.582 | 1 | 0.208 |
| [steroids=0] | 0^a^ | . | . | . | . | . | . | 0^a^ | . | . | . | . | . | . |
| [EEG=2] | -0.032 | 0.0370 | -0.105 | 0.040 | 0.769 | 1 | 0.381 | 1.034 | 0.4087 | 0.233 | 1.835 | 6.400 | 1 | 0.011 |
| [EEG=1] | 0.096 | 0.0564 | -0.015 | 0.206 | 2.873 | 1 | 0.090 | 1.124 | 0.4937 | 0.156 | 2.092 | 5.184 | 1 | 0.023 |
| [EEG=0] | 0^a^ | . |  |  | . | . | . | 0^a^ | . | . | . |  |  |  |
| (Scale) | 0.104 |  |  |  |  |  |  | 1 |  |  |  |  |  |  |

a. Set to zero because this parameter is redundant.

**C. Pairwise Comparisons**

| **Dependent Variable** | | **Expressive DQ** | | | | | | **Receptive DQ** | | | | | | | |
| --- | --- | --- | --- | --- | --- | --- | --- | --- | --- | --- | --- | --- | --- | --- | --- |
| **steroids** | **steroids** | Mean Difference | Std. Error | df | Sig. | 95% Wald CI Difference | | **steroids** | **steroids** | Mean Difference | Std. Error | df | Sig. | 95% Wald CI Difference | |
|  |  |  |  |  |  | Lower | Upper |  |  |  |  |  |  | Lower | Upper |
| Yes | No | 14.32 | 8.396 | 1 | 0.088 | -2.13 | 30.78 | Yes | No | 13.55 | 8.146 | 1 | 0.096 | -2.42 | 29.51 |
| No | Yes | -14.32 | 8.396 | 1 | 0.088 | -30.78 | 2.13 | No | Yes | -13.55 | 8.146 | 1 | 0.096 | -29.51 | 2.42 |
| **EEG** | **EEG** | Mean Difference |  | df | Sig. | 95% Wald CI Difference | | **EEG** | **EEG** | Mean Difference | Std. Error | df | Sig. | 95% Wald CI Difference | |
|  |  |  |  |  |  | Lower | Upper |  |  |  |  |  |  | Lower | Upper |
| EEG-2 | EEG-1 | -11.59 | 7.239 | 1 | 0.109 | -25.78 | 2.59 | EEG-2 | EEG-1 | -7.95 | 7.306 | 1 | 0.277 | -22.27 | 6.37 |
|  | EEG-0 | -12.29 | 6.007 | 1 | 0.041 | -24.07 | -0.52 |  | EEG-0 | -13.42a | 6.407 | 1 | 0.036 | -25.98 | -0.86 |
| EEG-1 | EEG-2 | 11.59 | 7.239 | 1 | 0.109 | -2.59 | 25.78 | EEG-1 | EEG-2 | 7.95 | 7.306 | 1 | 0.277 | -6.37 | 22.27 |
|  | EEG-0 | -0.70 | 5.365 | 1 | 0.896 | -11.22 | 9.82 |  | EEG-0 | -5.47 | 5.356 | 1 | 0.307 | -15.97 | 5.02 |
| EEG-0 | EEG-2 | 12.29 | 6.007 | 1 | 0.041 | 0.52 | 24.07 | EEG-0 | EEG-2 | 13.42a | 6.407 | 1 | 0.036 | 0.86 | 25.98 |
|  | EEG-1 | 0.70 | 5.365 | 1 | 0.896 | -9.82 | 11.22 |  | EEG-1 | 5.47 | 5.356 | 1 | 0.307 | -5.02 | 15.97 |

| **Dependent Variable** | | **Non-verbal IQ** | | | | | | **Extreme Behaviour** | | | | | | | | |
| --- | --- | --- | --- | --- | --- | --- | --- | --- | --- | --- | --- | --- | --- | --- | --- | --- |
| **steroids** | **steroids** | Mean Difference | Std. Error | df | Sig. | 95% Wald CI Difference | | **steroids** | **steroids** | Mean Difference | Std. Error | df | Sig. | 95% Wald CI Difference | | |
|  |  |  |  |  |  | Lower | Upper |  |  |  |  |  |  | Lower | Upper | |
| Yes | No | 0.08 | 2.585 | 1 | 0.975 | -4.98 | 5.15 | Yes | No | -0.09 | 0.079 | 1 | 0.234 | -0.25 | 0.06 | |
| No | Yes | -0.08 | 2.585 | 1 | 0.975 | -5.15 | 4.98 | No | Yes | 0.09 | 0.079 | 1 | 0.234 | -0.06 | 0.25 | |
| **EEG** | **EEG** | Mean Difference | Std. Error | df | Sig. | 95% Wald CI Difference | | **EEG** | **EEG** | Mean Difference | Std. Error | df | Sig. | 95% Wald CI Difference | | |
|  |  |  |  |  |  | Lower | Upper |  |  |  |  |  |  | Lower | Upper | |
| EEG-2 | EEG-1 | -11.13 | 4.331 | 1 | 0.010 | -19.62 | -2.65 | EEG-2 | EEG-1 | -0.02 | 0.110 | 1 | 0.837 | -0.24 | | 0.19 |
|  | EEG-0 | -2.69 | 2.852 | 1 | 0.346 | -8.28 | 2.90 |  | EEG-0 | 0.23 | 0.195 | 1 | 0.231 | -0.15 | | 0.62 |
| EEG-1 | EEG-2 | 11.13 | 4.331 | 1 | 0.010 | 2.65 | 19.62 | EEG-1 | EEG-2 | 0.02 | 0.110 | 1 | 0.837 | -0.19 | | 0.24 |
|  | EEG-0 | 8.44 | 4.951 | 1 | 0.088 | -1.26 | 18.15 |  | EEG-0 | 0.26 | 0.205 | 1 | 0.212 | -0.15 | | 0.66 |
| EEG-0 | EEG-2 | 2.69 | 2.852 | 1 | 0.346 | -2.90 | 8.28 | EEG-0 | EEG-2 | -0.23 | 0.195 | 1 | 0.231 | -0.62 | | 0.15 |
|  | EEG-1 | -8.44 | 4.951 | 1 | 0.088 | -18.15 | 1.26 |  | EEG-1 | -0.26 | 0.205 | 1 | 0.212 | -0.66 | | 0.15 |

CI: Confidence Interval; Std: Standard; Sig: Significance

## Supplementary Table 7: Phenotypic comparison between *GRIN2A*-positive (n=7) and *GRIN2A*-negative (n=38) individuals in study cohort

| Variant | FHx | Speech delay before regression | Age at Regression | Seizures | Language  Recovery | Below Ave non-verbal IQ | Behavioural difficulties | Motor Difficulties |
| --- | --- | --- | --- | --- | --- | --- | --- | --- |
| *GRIN2A* positive  Mean (range) or % | 57.1% | 71.4% | 6y0m ± 1y8m  (3y6m to 8y10m) | 42.9% | 100% | 57.1% | 57.1% | 57.1% |
| *GRIN2A* negative  Mean(range) or % | 39.5% | 23.7% | 4y9m ± 2y0m  (1y6m to 11y10m) | 89.5% | 82.2% | 42.9% | 84.2% | 23.7% |
| *p*- value  (<0.05: significant) | 0.433* | **0.023*** | 0.133† | **0.013*** | 0.569* | 0.699* | 0.131* | 0.168* |

Ave: Average; FHx: Family history; IQ: intelligence quotient; * Fisher’s exact test; †Independent T-test

## Supplementary Table 8: Clinical features for *GRIN2A*-positive LKS individuals reported in the literature

| **Case Identifier** | **Variant** | **FHx** | **Speech delay before SLR** | **Age at SLR/onset** | **Seizures** | **Language**  **Recovery** | **Below Ave non-verbal IQ** | **Behavioural difficulties** | **Motor Difficulties** |
| --- | --- | --- | --- | --- | --- | --- | --- | --- | --- |
| EA85^6^ | arr16p13.2;  16: 10,246,239-10,354,862 | Yes | Yes | 5y | Yes | No | No | Yes | NR |
| Family 3 301^7^ | arr16p13.2;  16:9,915,756-9,915,815 | Yes | No | 5y* | Yes | Yes | NR | No | NR |
| Case 1 DZ98^7^ | c.2797G>A; p.Asp933Asn | No | No | 1y* | Yes | Yes | No | No | NR |
| Case 3-81^7^ | c.2081T>C; p.Ile694Thr | No | No | 2y6m* | Yes | Yes | Yes | Yes | NR |
| Case 5 DY29^7^ | c.1642G>A; p.Ala548Thr | No | Yes | 6y* | Yes | Yes | Yes | Yes | NR |
| Family 1 DZ29^7^ | c.1123-2A>G | Yes | Yes | 4y* | Yes | Yes | Yes | No | NR |
| BIII2^8^ | c.2T>C; p.Met1Thr | Yes | No | 3y6m* | Yes | NR | Yes | NR | NR |
| BrnIndex-2^9^ | c.2041C>T; p.Arg681* | Yes | NR | 3y6m | No | NR | Yes | NR | No |
| BrnIndex-3^9^ | c.1007+1G>A | Yes | NR | 4y^†^ | Yes | NR | Yes | NR | No |
| Pt74-5^9^ | c.692G>A; p.Cys231Tyr | Yes | NR | 3y^†^ | Yes | NR | Yes | NR | Yes |
| Patient 7^10^ | c.1553G>A; p.Arg518His | No | NR | 6y | No | Yes | Yes | Yes | NR |
| Patient^11^ | c.2146G>A; p.Ala716Thr | Yes | No | 3y | Yes | Yes | NR | No | Yes |
| Family 1^12^ | c.2191G>A; p.Asp731Asn | Yes | NR | 5y^†^ | Yes | NR | NR | NR | NR |
| Patient 4^13^ | c.2407G>T; p.Glu803* | NR | Yes | 2y6m | Yes | Yes | Yes | NR | Yes |
| Patient 2^14^ | c.2041C>T; p.Arg681* | Yes | NR | 5y | Yes | Yes | Yes | Yes | Yes |

Ave: average; FHx: family history; SLR: speech and language regression; IQ: intelligence quotient; m: months; NR: not reported; y: years. *Only “age at seizure onset” reported, assumed to be close to age at SLR; ^†^only age at “onset” reported, assumed to be at/close to age at SLR.

## Supplementary Table 9: Clinical features for *GRIN2A*-negative LKS individuals reported in the literature

| **Case Identifier** | **FHx** | **Speech delay before SLR** | **Age at SLR/onset** | **Seizures** | **Language**  **Recovery** | **Below Ave non-verbal IQ** | **Behavioural difficulties** | **Motor Difficulties** |
| --- | --- | --- | --- | --- | --- | --- | --- | --- |
| 1-a^10^ | No | NR | 3y* | Yes | No | No | NR | NR |
| 2-a^10^ | No | NR | 5y* | Yes | Yes | Yes | NR | NR |
| 3^10^ | No | NR | 5y6m* | Yes | No | Yes | NR | NR |
| 4^10^ | No | NR | 3y* | Yes | No | Yes | NR | NR |
| 5^10^ | No | NR | 4y* | Yes | Yes | No | NR | NR |
| 6^10^ | Yes | NR | 4y6m* | Yes | Yes | Yes | NR | NR |
| 8^10^ | Yes | NR | 6y6m* | Yes | Yes | No | NR | NR |
| 9^10^ | No | NR | 2y6m* | Yes | No | No | NR | NR |
| 10^10^ | No | NR | 4y* | Yes | Yes | No | NR | NR |
| 11^10^ | No | NR | 6y6m* | Yes | Yes | No | NR | NR |
| 12^10^ | Yes | NR | 4y* | Yes | Yes | No | NR | NR |
| 13^10^ | No | NR | 5y* | No | Yes | No | NR | NR |
| 19^15^ | No | NR | 5y4m | Yes | NR | Yes | Yes | NR |
| 20^15^ | No | NR | 8y | No | Yes | No | Yes | NR |
| 21^15^ | Yes | NR | 7y9m | Yes | No | Yes | Yes | NR |

Ave: average; FHx: family history; SLR: speech and language regression; IQ: intelligence quotient; m: months; NR: not reported; y: years. *only age at “onset” reported, assumed to be close to age at SLR.

## Supplementary Table 10: Phenotypic comparison between *GRIN2A*-positive (n=22) and *GRIN2A*-negative individuals (n=53) – Study data combined with data from literature reports

| **Genotype** | **FHx** | **Speech delay before regression** | **Mean Age at**  **SLR/ Onset** | **Seizures** | **Language recovery** | **Below Ave non-verbal IQ** | **Behavioural. Difficulties** | **Motor difficulties** |
| --- | --- | --- | --- | --- | --- | --- | --- | --- |
| *GRIN2A* mutation positive  N= 22 | 66.7%  N^‡^= 21 | 56.3%  N^‡^= 16 | 4y7m ± 1y9m  (1y0m to 8y10m) | 72.7% | 94.1%  N^‡^=17 | 73.7%  N^‡^=19 | 56.3%  N^‡^= 16 | 61.5%  N^‡^= 13 |
| *GRIN2A* mutation negative  N= 53 | 35.8%  N=53 | 23.7%  N^‡^= 38 | 4y10m ± 1y11m  (1y6m to 11y10m) | 88.7% | 78.8%  N^‡^=52 | 45.3%  N=53 | 85.4%  N^‡^= 41 | 23.7%  N^‡^= 38 |
| *p*- value  (<0.05: significant) | **0.021*** | **0.029*** | 0.659† | 0.163* | 0.269* | 0.059* | **0.033*** | **0.019*** |

FHx: Family history; *Fisher’s exact test; †Independent T-test; IQ: intelligence quotient; SLR: speech and language regression; N: number of patients; N^‡^: where data is missing, this indicates the number of patients with data available for analysis

# Supplementary Figure 1

BL: Beta-lactamase assay; Ca^2+^ influx Ix: Ca^2+^ influx imaging; CTD: carboxyl-terminal domain; HC: HEK293 cells; IC: immunocytochemistry; M1-M4: transmembrane domains; NTD: N-terminal domain; S1 and S2: subunits of ligand-binding domain; SCR: single-channel recording; TEVC: two-electrode voltage clamp; WB: Western blot; WCPC: whole-cell patch clamping, XO: *Xenopus laevis* oocytes.

★: Landau Kleffner Syndrome; ◆Self-limiting epilepsy with centrotemporal spikes; 🞆 other epileptic encephalopathies with spike-wave activation in sleep (EE-SWAS).

# Supplementary References

1. Mestek-Boukhibar L, Clement E, Jones WD, et al. Rapid Paediatric Sequencing (RaPS): comprehensive real-life workflow for rapid diagnosis of critically ill children. *J Med Genet* 2018;55(11):721-728. doi: 10.1136/jmedgenet-2018-105396

2. Chen X, Keramidas A, Harvey RJ, Lynch JW. Effects of GluN2A and GluN2B gain-of-function epilepsy mutations on synaptic currents mediated by diheteromeric and triheteromeric NMDA receptors. *Neurobiol Dis* 2020;140:104850. doi: 10.1016/j.nbd.2020.104850

3. Schmittgen TD, Livak KJ. Analyzing real-time PCR data by the comparative C(T) method. *Nat Protoc* 2008;3(6):1101-8. doi: 10.1038/nprot.2008.73

4. Dixon CL, Zhang Y, Lynch JW. Generation of functional inhibitory synapses incorporating defined combinations of GABA_A_ or glycine receptor subunits. *Front Mol Neurosci* 2015;8:80. doi: 10.3389/fnmol.2015.00080

5. Robinson RO, Baird G, Robinson G, Simonoff E. Landau-Kleffner syndrome: course and correlates with outcome. *Dev Med Child Neurol* 2001;43(4):243-7. doi: 10.1017/s0012162201000469

6. Lesca G, Rudolf G, Labalme A, et al. Epileptic encephalopathies of the Landau-Kleffner and continuous spike and waves during slow-wave sleep types: genomic dissection makes the link with autism. *Epilepsia* 2012;53(9):1526-38. doi: 10.1111/j.1528-1167.2012.03559.x

7. Lesca G, Rudolf G, Bruneau N, et al. *GRIN2A* mutations in acquired epileptic aphasia and related childhood focal epilepsies and encephalopathies with speech and language dysfunction. *Nat Genet* 2013;45(9):1061-6. doi: 10.1038/ng.2726

8. Carvill GL, Regan BM, Yendle SC, et al. *GRIN2A* mutations cause epilepsy-aphasia spectrum disorders. *Nat Genet* 2013;45(9):1073-6. doi: 10.1038/ng.2727

9. Lemke JR, Lal D, Reinthaler EM, et al. Mutations in *GRIN2A* cause idiopathic focal epilepsy with rolandic spikes. *Nat Genet* 2013;45(9):1067-72. doi: 10.1038/ng.2728

10. Conroy J, McGettigan PA, McCreary D, et al. Towards the identification of a genetic basis for Landau-Kleffner syndrome. *Epilepsia* 2014;55(6):858-65. doi: 10.1111/epi.12645

11. Fainberg N, Harper A, Tchapyjnikov D, Mikati MA. Response to immunotherapy in a patient with Landau-Kleffner syndrome and *GRIN2A* mutation. *Epileptic Disord* 2016;18(1):97-100. doi: 10.1684/epd.2016.0791

12. Dyment DA, Tetreault M, Beaulieu CL, et al. Whole-exome sequencing broadens the phenotypic spectrum of rare pediatric epilepsy: a retrospective study. *Clin Genet* 2015;88(1):34-40. doi: 10.1111/cge.12464

13. von Stulpnagel C, Ensslen M, Moller RS, et al. Epilepsy in patients with *GRIN2A* alterations: Genetics, neurodevelopment, epileptic phenotype and response to anticonvulsive drugs. *Eur J Paediatr Neurol* 2017;21(3):530-541. doi: 10.1124/mol.116.10678110.1016/j.ejpn.2017.01.001

14. Hausman-Kedem M, Menascu S, Greenstein Y, Fattal-Valevski A. Immunotherapy for *GRIN2A* and *GRIN2D*-related epileptic encephalopathy. *Epilepsy Res* 2020;163:106325. doi: 10.1016/j.eplepsyres.2020.106325

15. Pavlidis E, Møller RS, Nikanorova M, et al. Idiopathic encephalopathy related to status epilepticus during slow sleep (ESES) as a "pure" model of epileptic encephalopathy. An electroclinical, genetic, and follow-up study. *Epilepsy Behav* 2019;97:244-252. doi: 10.1016/j.yebeh.2019.05.030.

16. Addis L, Virdee JK, Vidler LR, Collier DA. Epilepsy-associated *GRIN2A* mutations reduce NMDA receptor trafficking and agonist potency - molecular profiling and functional rescue. *Sci Rep* 2017;7(1):66. doi: 10.1038/s41598-017-00115-w

17. Sibarov DA, Bruneau N, Antonov SM, Szepetowski P, Burnashev N, Giniatullin R. Functional properties of human nmda receptors associated with epilepsy-related mutations of GluN2A subunit. *Front Cell Neurosci* 2017;11:155. doi: 10.3389/fncel.2017.00155

18. Swanger SA, Chen W, Wells G, et al. Mechanistic insight into NMDA receptor dysregulation by rare variants in the GluN2A and GluN2B agonist binding domains. *Am J Hum Genet* 2016;99(6):1261-1280. doi: 10.1016/j.ajhg.2016.10.002.

19. Gao K, Tankovic A, Zhang Y, et al. A *de novo* loss-of-function *GRIN2A* mutation associated with childhood focal epilepsy and acquired epileptic aphasia. *PLoS One* 2017;12(2):e0170818. doi: 10.1371/journal.pone.0170818.

20. Shepard N, Baez-Nieto D, Iqbal S, et al. Differential functional consequences of *GRIN2A* mutations associated with schizophrenia and neurodevelopmental disorders. *Sci Rep* 2024;14(1):2798. doi: 10.1038/s41598-024-53102-3.

21. Xie L, McDaniel MJ, Perszyk RE, et al. Functional effects of disease-associated variants reveal that the S1-M1 linker of the NMDA receptor critically controls channel opening. *Cell Mol Life Sci* 2023;80(4):110. doi: 10.1007/s00018-023-04705-y.
